# *In vitro* screening of topical formulation excipients for epithelial toxicity in cancerous and non-cancerous cell lines

**DOI:** 10.17179/excli2023-6072

**Published:** 2023-11-16

**Authors:** Farzaneh Forouz, Yousuf Mohammed, Hamid S. A. Shobeiri Nejad, Michael S. Roberts, Jeffrey E. Grice

**Affiliations:** 1 Therapeutics Research Group, Frazer Institute, The University of Queensland, Woolloongabba, QLD 4102, Australia; 2 School of Pharmacy, The University of Queensland, Woolloongabba, QLD 4102, Australia; 3 School of Mathematics, Physics and Computing, University of Southern Queensland, QLD 4350, Australia; 4 School of Pharmacy and Medical Sciences, University of South Australia, Adelaide, SA 5000, Australia; 5 Therapeutics Research Centre, Basil Hetzel Institute for Translational Medical Research, The Queen Elizabeth Hospital, Woodville, SA 5011, Adelaide, Australia

**Keywords:** pharmaceutical excipients, cytotoxicity cell sensitivity, MTT, crystal violet, ROS, cell cycle analysis

## Abstract

Chemical excipients used in topical formulations may be toxic to living skin cells. Here, we compared the *in vitro* toxicity of some common solubilizing excipients against human melanoma cells, human keratinocytes (HaCaT) and primary skin fibroblasts (FB) as examples of cancerous, immortalized and primary human skin cells, often used as experimental models representative of *in vivo* conditions. Two distinct endpoint assays (3-(4,5-Dimethylthiazol-2-yl)-2,5-diphenyltetrazolium bromide (MTT) and crystal violet (CV)) were used. The mechanism of cell death after excipient exposure was assessed through Reactive Oxygen Species (ROS) production, cell membrane integrity and cell cycle progression. Results showed that the surfactants, Labrasol®, Labrafil® and Transcutol®, were less toxic than Triton X-100 (a model irritant) in all cell types whereas the oil, Labrafac®, was non-toxic. The human melanoma WM164 cell line showed the greatest sensitivity toward cytotoxicity after chemical exposure, while the other cell lines were more resistant. The relative excipient cytotoxicity responses observed in the MTT and CV assays were comparable and similar trends were seen in their estimated 50 % inhibitory concentration (IC_50_) values. DNA fragmentation by flow cytometry after exposing the cells to IC_50_ concentrations of the excipients showed negligible apoptotic populations. ROS production was increased in all cell types after toxic exposure; however, ROS elevation did not lead to apoptosis. The toxicity profiles of each excipient are not only relevant to their use in formulating safe topical products but also in the potential synergistic efficacy in the topical treatment of melanoma.

## Introduction

Products applied to the skin for therapeutic, cosmetic, or other purposes must be rigorously assessed for safety (FDA, 2020[29]), particularly for adverse effects such as skin irritation and allergic reactions. Toxicity may be due to direct effects of formulation ingredients or be caused by degradation products following processes initiated by heat (Nguyen et al., 2022[61]), pH (Das and Wong, 2020[22]) or light (Ioele et al., 2021[42]; Kowalska et al., 2021[49]; Kryczyk-Poprawa et al., 2019[50]). The initiation of toxic degradation products by light exposure is a major issue in skin formulations and can affect multiple components, including actives and excipients (inactive ingredients that are included to meet the essential physical, microbiological, chemical and biopharmaceutical needs of the final formulation). Furthermore, other factors including the wavelength and intensity of light, pH, temperature, and concentration can affect the outcome of the degradation process (Kryczyk-Poprawa et al., 2019[50]). It is recommended that all ingredients in products applied to the skin, including excipients, be tested for photostability (Kowalska et al., 2021[49]). On the other hand, excipients are commonly selected to protect active components from the effects of photodegradation and limit phototoxicity (Baertschi et al., 2015[8]; Das and Wong 2020[22]; Ioele et al., 2021[42]; Kryczyk-Poprawa et al., 2019[50]). 

The potential effects of topical excipients, including skin irritation and allergic reactions (Charmeau-Genevois et al., 2021[19]; Coloe and Zirwas, 2008[21]; Osterberg and See, 2003[66]), and their impacts on therapeutic skin target cells (Kalasz and Antal, 2006[43]; Nogueira et al., 2011[62]), whether desirable or undesirable, have received relatively less attention, compared to the effects of active drugs. This work concentrates on that aspect by examining the direct effects of four of the most commonly used topical excipients.

An understanding of both the mechanisms of action and the toxicity profiles of excipients is important during formulation development and general safety evaluation of a product. Skin irritation and sensitization have now been described for a range of topical product excipients, including the skin irritancy of solvents, mineral oils and surfactants (Barbaud et al., 2011[10]; Patrick et al., 1985[67]). Of particular interest are surfactants, the most commonly used excipients in topical formulations (Kumar Sharma et al., 2021[51]), where they act as solubilizers of lipophilic compounds and stabilizers of multiphase systems that enhance the topical bioavailability of active compounds. As well as being irritant, topical surfactants may also lead to skin dryness and itchiness (Bárány et al., 1999[9], 2003).

Generally, the extent of skin irritation caused by a given surfactant is related to its concentration and physicochemical properties. Anionic surfactants are widely recognized as strong irritants of human and animal skin and sodium dodecyl sulphate (SDS) is broadly applied as a model irritant in skin toxicity investigations. On the other hand, non-ionic surfactants are considered to have the least irritant potential, making them a major class of excipients used in pharmaceutical products (Effendy and Maibach 1995[27]). SDS is used in topical formulations to increase skin penetration of an active drug, however the safe concentrations should be considered (Aungst et al., 1986[6]). Labrasol®, Labrafil® and Transcutol® are excipients incorporated as solvents or co-solvents, separately or in combination, to enhance penetration of topical drug formulations (Fini et al., 2008[33]; Senyiğit et al., 2009[81]; Zhao et al., 2011[94]). These compounds cannot be considered as non-toxic excipients in pharmacology or toxicology assessments when administered to the human body through different routes (Budden et al., 1979[16]). 

Currently, the mode of action by which surfactants cause skin irritation is not completely understood. In general terms, possible mechanisms include (i) a direct impact on lipids and proteins of the stratum corneum, (ii) action on living cells of the epidermal layer to cause cell death or change their proliferative capability and (iii) interaction with various components of the dermal layer to release mediators causing inflammatory responses (Rhein et al., 1990[74]). A range of mechanisms has been invoked for direct cytotoxicity caused by surfactants or other toxic substances, based on the different *in vitro* end point assays and toxicants used. It is unlikely that a single mechanism of cytotoxicity will apply in all cases and more research using different classes of toxicants, cell types and standardized end point assays is required. Cytotoxic responses may result from interaction with a target molecule to affect gene transcription and expression during signal transduction, leading to cellular dysfunction. Significant alterations in normal cell functioning can cause cell injury or death (Gregus, 2008[38]). Xenobiotics could activate signalling pathways from the receptors located on cell membrane to transcription factors in the nuclease influencing transcription of the genes that are responsible for regulation of cell cycle. Some signals activate cell cycle arrest resulting in apoptosis (Gregus, 2008[38]). 

Cytotoxic responses can also be exploited for cancer therapy, which can work through two methods of action; by stimulation of apoptosis and by direct toxicity (Gerl and Vaux, 2005[36]; Pfeffer and Singh, 2018[71]). Therefore, information about cell death regulation and the cell cycle checkpoint is important in developing therapeutic agents and cancer treatments. Presently, the influence of cell cycle checkpoint regulation to induce synergistic cell death enhancement is unidentified. Therefore, additional study on checkpoint retraction and checkpoint arrest, as a mean to improve the cytotoxic effects of chemotherapy is required (Tyagi et al., 2002[88]). 

To comply with the topical drug safety rules, pre-clinical safety evaluation studies are required (Pugsley et al., 2008[72]). A common strategy is to collect toxicity information of all ingredients, including inactive excipients, before incorporating them into the final products for human use applications (Fischer et al., 2003[34]; Nogueira et al., 2011[62]). Previously, dermal toxicity was evaluated by monitoring the effects of exposure to a test chemical on the skin of animals, commonly known as the Draize skin irritation test (Draize, 1944[24]). However, from 2009 only *in vitro* tests have been permitted in the European Union to validate the safety of new cosmetic ingredients for the skin applications (EC, 2009[26]). Ethical issues and regulatory difficulties associated with animal testing, in addition to some disadvantages of animal models have encouraged researchers to develop alternative *in vitro* methods for predicting potential hazardous effects of chemical permeants for scientific and industrial applications (Robinson et al., 2002[75]). Much of the works have been towards a new approach more strongly justified in human biology such as measuring the intrinsic cytotoxic effects of pure chemicals, mixtures and formulations on cells in culture (Andersen and Krewski, 2009[5]; Goldberg and Frazier, 1989[37]). Since the reliance on a single assay can be associated with a risk of inaccurate interpretation, a battery of methods based on different mechanisms is usually performed simultaneously to evaluate the toxic (and presumably) irritant effects of chemical compounds on cultured cells as an alternative to animal tests (Benavides et al., 2004[12]; Osborne and Perkins, 1991[65]; Sanchez et al., 2006[77]).

Several end-point assays for testing cytotoxicity in cell culture are available. The assessment of cell proliferation rate as a measure of cell viability (Martinez et al., 2006[55]) is commonly performed using the 3-(4,5-Dimethylthiazol-2-yl)-2,5-diphenyl tetrazolium (MTT) assay, which is based on the mitochondrial activity of living cells resulting in reduction of the yellow tetrazolium salt MTT to insoluble purple formazan crystals (Mosmann, 1983[59]). Crystal violet (CV) staining is another cytotoxicity assay that is used to assess the proliferation rate of cells after exposure to chemical substances by measuring the total DNA mass of viable cells that adhere to the cell culture plate (Feoktistova et al., 2016[32]). The CV assay was shown to provide reliable results in studying the effects of chemotherapeutics or other compounds on cell survival and growth inhibition of cancer cell lines (Emran et al., 2018[28]). In many cancer cells, reactive oxygen species (ROS) are produced by mitochondria as a response to toxicity and overproduction of ROS can carry cells into oxidative stress, causing damage to cellular proteins, DNA and lipid elements and consequently increasing cell death cells (Perillo et al., 2020[69]). Intercellular ROS can be detected by the use of an uncharged and non-fluorescent probe, Dihydrorhodamine 123 (DHR). DHR can passively enter cells where it is oxidized by mitochondrial ROS to produce Rhodamine 123 (R123), a positively charged, green fluorescent dye that can then be detected as it accumulates in the mitochondria (Kiani-Esfahani et al., 2012[45]). Finally, toxicity can be assessed *in vitro* by evaluating the cell membrane permeability based on the uptake of dyes such as trypan blue. Trypan blue penetrates and stains dead cells a dark blue color when cellular membrane integrity is compromised, while viable cells remain unstained with a clear appearance surrounded by a refractile ring. This method only distinguishes live from dead cells and cannot differentiate between healthy cells and cells that are alive but have impaired cellular function (metabolically inactive) (Stoddart, 2011[85]). 

As noted above, *in vitro* cell culture assays can provide useful pre-clinical information about the cytotoxicity of substances, including formulation excipients. However, in order for the results to be applicable to humans, a direct correlation between *in vitro* and *in vivo* findings is required. In the absence of such a correlation, *in vitro* cell culture assays may still be useful for screening purposes (Dhawan and Kwon. 2018[23]). A number of studies have found cell culture to be suitable as a primary model for establishing the dermal irritancy level of chemical substances. For example, irritancy screening for valproate performed in HaCaT cell culture gave comparable results to those from subsequent skin patch testing (Choi et al., 2013[20]). 

Sanchez et al., used cell viability measurement on HaCaT cells as an *in vitro* model to predict the potential skin irritation of several pharmaceutical excipients, including surfactants (Sanchez et al., 2006[77]), while Korting studied the irritancy potential of surfactants using *in vitro* cytotoxicity assays based on human keratinocytes, HaCaT cells and mouse fibroblasts and compared the results to those from *in vivo* soap chamber assays (Korting et al., 1994[48]). Overall, it is believed that using *in vitro* systems to detect primary cellular responses may assist to predict toxic responses *in vivo*. 

An early study measured the cytotoxicity of substances on human cell cultures by different endpoint assays and compared the results with the human skin patch test. The results indicated a close correlation of the dose-responses for the endpoint assays and the human patch test following exposure to the tested chemicals. These outcomes illustrate the validity of human skin cell cultures and of cell viability, and cytotoxicity as endpoints, for the *in vitro *assessment of skin irritancy (Osborne and Perkins, 1994[64]). Later on, other studies developed models to compare the responses from toxicity *in vitro* and skin *in vivo* with good correlation (Benassi et al., 2003[11]; Wilhelm et al., 2001[93]). Another study assessed the skin irritation potential of surfactants in *in vitro* human skin fibroblast cells by Alamar Blue and *in vivo* by the human skin patch test. A close relationship was seen between the cellular Alamar Blue assay and the human patch test, with r=0.867. These results supported the proposal that *in vitro* end point assays could be used to predict the irritancy level of various surfactants in humans (Lee et al., 2000[52]).

The goal of the present work was to investigate the potential cytotoxicity of some common pharmaceutical excipients, chosen previously as ingredients in self-emulsifying microemulsion formulations in an ongoing program to develop for effective topical anti-cancer treatments (Forouz et al., 2020[35]), and to compare the sensitivity of two endpoint measurement methods in cancer and non-cancer cell lines. In doing so, we exploited our knowledge of mechanisms involved in exerting toxicity and cell death after treatment by excipients in the tested cell lines. The toxic effects of these excipients, known to have various enhancement properties for topical delivery, were investigated on three different human melanoma cell lines and two normal human cell lines (keratinocytes and primary fibroblasts) by measuring cell viability using two different biological assays. To learn more about the mechanism of cell death induced by the excipients, cell membrane integrity was tested, reactive oxygen species (ROS) was measured, and cell cycle progression was analyzed.

## Materials and Methods

### Chemical and reagents

3-(4,5-Dimethylthiazol-2-yl)-2,5-diphenyl tetrazolium (MTT), crystal violet solution, Triton X-100, and dimethylsulphoxide (DMSO) were purchased from Sigma-Aldrich (St. Louis, MO, USA). RPMI 1640, Dulbecco's modified Eagle's medium (DMEM), and fetal bovine serum (FBS), phosphate buffered saline (PBS), trypsin-EDTA solution (170,000 U/l trypsin and 0.2 g/l EDTA), and penicillin-streptomycin solution (10,000 U/ml penicillin and 10 mg/ml streptomycin) were purchased from Gibco® (Australia). The culture flasks and multiple-well plates were from Corning® Costar®. All the reagents and solvents were analytical grade.

### Surfactants and other tested excipients

Labrasol® (caprylocaproyl macrogol-8- glyceride/ Caprylocaproyl Polyoxyl-8 glycerides), Labrafil® M 1944 CS (oleoyl polyoxyl-6 glycerides/ Oleoyl macrogol-6 glycerides), Transcutol® CG (diethylene glycol monoethyl ether), Labrafac® (Propylene glycol dicaprylocaprate). All these excipients were purchased from Gattefosse (St. Priest, France). Propylene glycol and Ethanol were purchased from Sigma-Aldrich (St. Louis, MO, USA). All excipients used as procured without further purification. A list of the tested excipients with some of their physicochemical information are demonstrated in Table 1(Tab. 1).

### Cell cultures

Human melanoma cell lines (WM164, WM1366, and D24), spontaneously immortalized normal human keratinocytes (HaCaT), and primary skin fibroblast cells were provided kindly by Prof. Helmut Schaider laboratory (UQ/Frazer Institute). The cancerous cell lines and their mutation are listed in Table 2(Tab. 2). The cells were grown in RPMI medium (+ L-glutamine) supplemented with 5 % (v/v) FBS, 2 % penicillin/streptomycin except for the fibroblast cells which were cultured in DMEM. Cells were routinely grown in culture flasks and maintained at 37 °C in a humidified 5 % CO_2 _atmosphere. Microbial contamination test was performed routinely in our laboratory. Cells were trypsinized using trypsin-EDTA when they reached approximately 80 % confluence to co-culture or seed.

### Experimental design

Cells were seeded into the 96-well cell culture plates in 100 μl of complete culture medium at the initial density of 2x10^4^ (cells/ml). Cells were incubated under 5 % CO_2 _at 37 °C for 24 hours for adhering to the wells and medium was then replaced with 100 μl of fresh medium containing excipient solution at the required final concentration. Each concentration was tested in minimum of 5 replicates and control cells were exposed to the culture medium only.

### Cytotoxicity assays

#### MTT assay for assessing cell viability

After cell incubation for 24 h, the excipient-containing medium was removed, and 100 μl of MTT in PBS (5 mg/ml) diluted 1:10 in medium was then added in the dark. Plates were further incubated for 2-3 h, after which time the medium was removed and the purple formazan product was dissolved by adding 200 μl of DMSO to each well. Plates were then placed in a shaker for 5 min at room temperature and the absorbance of the resulting solutions was measured at 570 nm using a microplate reader (Multiskan™ FC Microplate Photometer). The effect of each treatment was calculated as the percentage of tetrazolium salt reduction by viable cells against the negative control (cells treated with culture medium only). 

#### Crystal violet assay (CV) for assessing cell viability

Fixed cells were stained with crystal violet dye solution to screen cell viability under treatment conditions. Cells are fixed by 4 % (v/v) paraformaldehyde in PBS for 10 minutes. Crystal violet assay of 0.05 % (v/v) is prepared by 1:4 dilution of the crystal violet stock solution (v/v) in 4 % (v/v) paraformaldehyde. The fixed cells were washed twice with PBS and covered with 250 µl crystal violet working solution. The plates were placed on a bench rocker with medium frequency (20 oscillations per minute) at room temperature for 20 minutes. The plates were emptied and washed under indirect stream of tap water for three times and inverted on filter paper. The plates were taped gently on the paper to remove any remaining liquid and air dried with no lid for minimum of 2 hr at room temperature. After the plates were photographed, blue dye was dissolved in DMSO and the optical density of each well was measured at 570 nm (OD570) with a plate reader (Multiskan™ FC Microplate Photometer). The effect of each treatment was calculated as the percentage of crystal viable staining of surviving attached cells, against the negative control (cells treated with culture medium only).

### Evaluation of membrane integrity by trypan blue exclusion assay

The dye exclusion test is routinely applied in laboratories to assess the number of live cells present in a cell suspension. Since viable cells retain intact cell membranes and exclude specific dyes, such as trypan blue, whereas dead cells do not. In this assay a cell suspension is mixed with dye and then visualized to distinguish the cells that take up dye from the ones that exclude it. Viable cells will be seen as a clear cytoplasm whereas a dead cell will appear with a blue cytoplasm (Strober, 2001[86]). After cell incubation for 24 h, the excipient-containing medium was removed, and cells were trypsinized using trypsin-EDTA to obtain cell suspension. 20 µl of trypan blue dye was mixed thoroughly with 20 µl cell suspension by pipetting ups and downs and loaded into the cell counting slide. Cell numbers were detected by the automated cell counting machine. To find the total number of viable cells in one ml of aliquot, the number of viable cells was multiplied by 2 (the dilution factor for trypan blue). Viable cell percentage was calculated as follows:


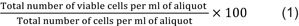


### Reactive Oxygen Species evaluation with dihydrorhodamine 123

The oxidant-sensitive dye, dihydrorhodamine 123 (DHR), was used to detect intracellular ROS level. Cells were plated in the flat bottom 96-well plates to sub confluent (60-70 %) then the medium was removed and replaced with the pre-warmed treatment solutions (medium containing different concentration of the excipients). After 24 hours incubation with treatment solutions, medium was removed and replaced with the 100 μl of pre-warmed dying buffer (Hank's balanced salt solution (HBSS) containing 12 µM DHR dye). Cells were incubated for 30 minutes at 37 °C in a humidified 5 % CO_2_ atmosphere in dark. The cellular fluorescence generated by the DHR oxidation was measured at 485 nm (OD 485) using a microplate reader (Multiskan™ FC Microplate Photometer). Hydrogen peroxide (H_2_O_2_) treatment (800, 400, 200, and 100 µM)/ (27, 13.5, 6.75, 3.4 µg/ml) was used as positive control and dying buffer with no cells as blank control. Data points were recorded every 10 minutes for 60 minutes and exported for analysis by Excel software. The ROS level is calculated as the difference between (Ftest/ Fcontrol) and expressed as percentages of the control value using this formulation:


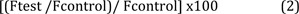


where *F_test_* and *F_control_* are the fluorescence intensities of the cells exposed to the treatments and the cells with only medium, respectively. Fluorescence intensity of the blank wells were measured at every measurement time points and recorded to compare with the control wells.


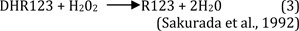


### Determination of DNA content and cell cycle analysis 

Each phase of the cell cycle was evaluated by DNA flow cytometry analysis. Cells were cultured in 6-well plates to sub confluent (60-70 % confluent) and then treated with IC_50_ concentrations of the excipients in each cell line for 24 hours. The PI staining was used to determine the DNA content and the cell-cycle phase distributions. Attached and floating cells were collected and fixed followed by PI staining and analyzed with FACs machine according to the instructions of the manufacturer. The percentages of cells in each cell-cycle phase were analyzed using FlowJo software (v10.6.1).

### Statistical analysis

To assess the excipients' cytotoxicity, the dose-response effect of each excipient was measured against the cell lines. The cytotoxicity value for each concentration of the excipients was expressed as percentage of viability against the untreated control wells (the optical density of untreated cells was set at 100 % viability) to construct dose-response curves. Dose-response curves from 24-h exposure of the cell lines to Labrasol®, Labrafil®, Transcutol®, and Labrafac® were plotted to show cell viability ( %) vs log of treatment concentration (µg/ml). In the present study, all cytotoxicity experiments of the excipients were performed in minimum of 5-6 replicates and results were displayed as mean ± standard deviation (SD). 

The cytotoxicity of the excipients was expressed in terms of an IC_50 _values (concentration causing 50 % death of the cell population) (Goldberg and Frazier, 1989[37]). IC_50_-value for each excipient was calculated from the best-fit (R2>0.95) of the Hill slope curve to experimental data using nonlinear regression analysis in Graph Pad Prism (Version 8.3.0, GraphPad Software, Inc., La Jolla, USA), based on the formula: Y = 100/1+10^((LogIC_50_-X)*Hillslope)) where X = log of dose, Y = growth inhibition value normalized to control, and Hillslope = unitless slope factor or Hill slope. All IC_50_ values were expressed as µg/ml. The percentage viability was calculated as follows:


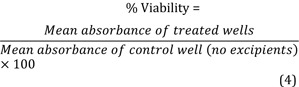


Statistical analyses were performed using ordinary one-way analysis of variance (ANOVA) test to determine the significant differences between the MTT and CV assays as well as differences between OD reading results of the treated and untreated control obtained from MTT and CV assays using Graph Pad Prism (Version 8.3.0, GraphPad Software, Inc., La Jolla, USA). Sidak's and Bartlett's post hoc analysis were applied with ANOVA test when they were appropriate. The non-parametric Kruskal-Wallis with Dunn's test was used to detect significant differences between the IC_50 _of the excipients in all cell lines obtained by TB, MTT, and CV assays.

## Results

### In vitro cytotoxicity of excipients

**Figure 1:**
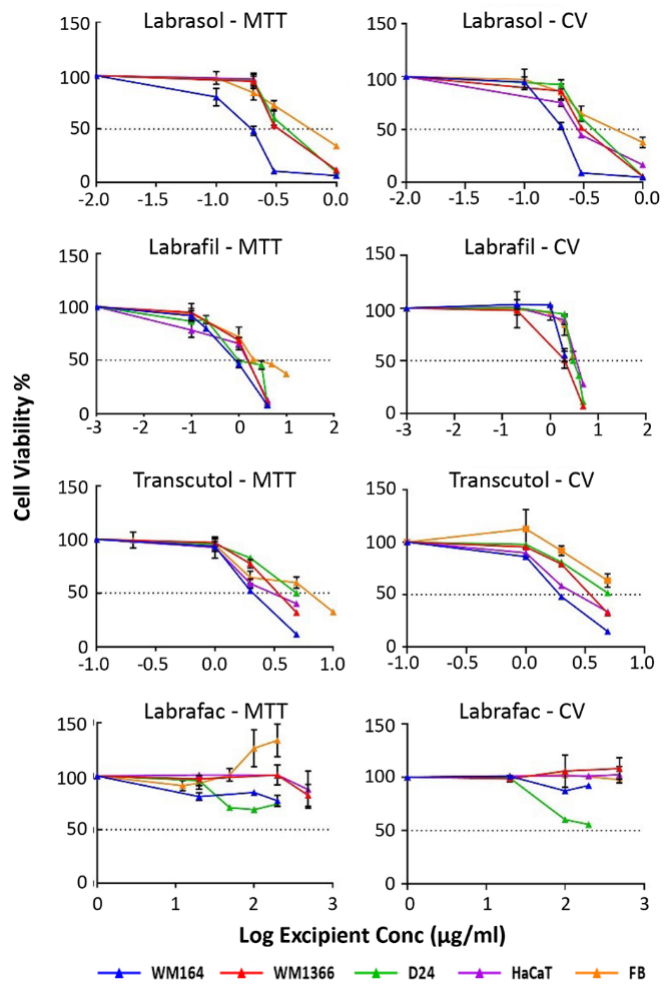
Representative dose-response curves from 24-h exposure of the tested cell lines to the tested excipients. Labrasol®, Labrafil®, Transcutol®, and Labrafac®. Values measured by MTT and CV assays. Data are expressed as mean ± SD of minimum 5-6 replicates.

The cytotoxic properties of the excipients were evaluated by cell viability responses in MTT and CV assays. Dose-response curves obtained from cytotoxicity assays following 24 h exposure to the cancerous and non-cancerous cell lines are illustrated in Figure 1(Fig. 1). The plots show cell viability % (y) values versus log of the concentration (µg/ml) (x) obtained from MTT and CV assays. The applied concentrations of the excipients for MTT and CV were selected from a wide range of concentrations to show the cellular responses to toxicity for each tested excipient.

Labrasol®, Labrafil®, and Transcutol® showed cell viability reduction effects within the applied concentration ranges in all cell lines but Labrafac® did not induce any reduction in cell viability at the applied concentrations.

**Figure 2:**
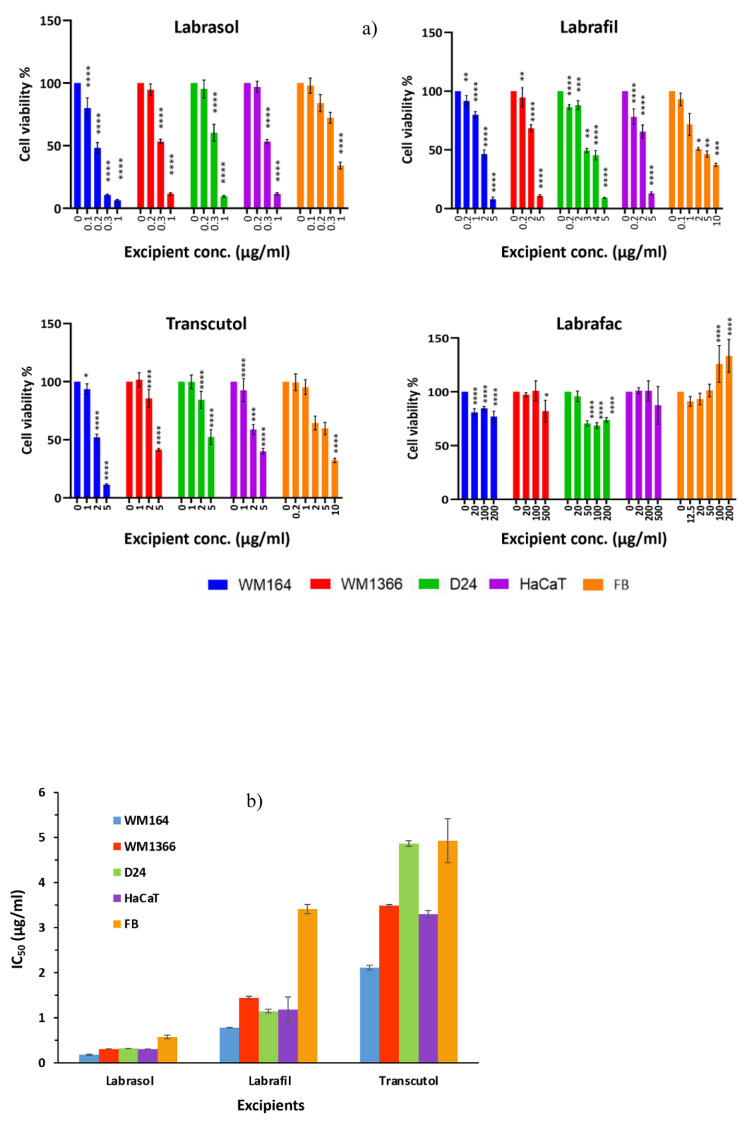
a) Cell viability % obtained from MTT assay and calculated as percentage of untreated control data are presented as mean±SD (n=minimum 5). Data were analyzed using One-way ANOVA with Bartlett's post hoc test. (p values of **** <0.0001; *** <0.001; ** <0.01; * <0.5) denote significant differences from the control value. (b) Cytotoxicity of the excipients expressed as IC_50_ values (µg/ml) on melanoma (WM164, WM1366, and D24) and Normal (HaCaT and FB) cells and measured by MTT assay. (MTT and CV were found to be significantly correlated assays hence having similar outcome). For each excipient WM164 is the most sensitive cancerous cell line and normal FB is the most resistant one. Labrafac® was excluded because it caused no toxicity to any cell lines at the concentrations used in this study.

Figure 2a)(Fig. 2) shows bar graphs of excipient concentrations plotted against the cell viability % after 24-hr exposure to the excipients. The raw data obtained from the optical density (OD) reading of each treatment well by MTT assay was compared to the untreated control well and statistically significant differences from the control were evaluated. One-way ANOVA followed by Bartlett's test was selected considering the equal variance across the group sample. Figure 2b)(Fig. 2) illustrates cytotoxicity of the excipients presented as IC_50_ values (µg/ml) on melanoma and normal cells, derived from MTT assay. Results derived from MTT and CV assays were found to be significantly correlated.

The sensitivities of the different cell lines to the excipients, assessed by MTT and CV assays, were expressed as half maximal inhibitory concentrations (IC_50_ (µg/ml)), where the lowest IC_50_ value indicates the most cytotoxic excipient. IC_50_ values were compared to those from Triton X-100, a highly irritant non-ionic surfactant that was employed as a positive control.

As shown in Table 3(Tab. 3), the cytotoxicities of excipients, determined by either the MTT or CV assay, were ranked in the order Triton-X-100 > Labrasol® > Labrafil® > Transcutol® > Labrafac® for all cell lines with the exception of Labrafil® and Transcutol® in HaCaT cells. The positive control, Triton X-100, was the most cytotoxic surfactant tested, causing significant inhibition of cell growth in all cell lines with exposure to 0.004 µg/ml concentration. Both tested surfactants, Labrasol® and Labrafil® showed less cytotoxic effects than the reference Triton X-100, predicting less irritancy effects *in vivo*. No damaging effects on cell growth were seen with Labrafac® in any cell line (Table 3(Tab. 3)). These results highlight the differences in sensitivities of all cell lines to the same excipient. The cancerous, WM164 was the most sensitive cell line to every excipient tested, whereas normal human fibroblasts were the least sensitive. For example, IC_50_ values for Labrasol® in WM164 was less than 0.2 µg/ml, compared to 0.62 µg/ml in primary fibroblast cells, indicating the greater sensitivity of the WM164 cancerous cell line over normal human fibroblasts. 

In general, IC_50 _values derived from the assays showed that the normal keratinocytes and the primary fibroblasts were more resistant to the toxicity of the tested excipients than the cancerous cell lines. Of these normal cell types, the primary fibroblasts were more resistant to the toxicity effect of the excipients than the keratinocytes (Table 3(Tab. 3)). The WM1366 and D24 cancerous cell lines were more resistant to the damaging effects of the excipients while the WM164 cell line showed greater sensitivity to all excipients. Labrafac® caused no toxicity to any cell lines at the concentrations used in this study.

### Comparison of MTT and CV cytotoxicity assays

To investigate the effect of the test methods on the calculated viability, the cell viability percentages for each excipient derived from MTT and CV assays were compared and analyzed. One-way ANOVA analysis followed by Sidak's post hoc test were used to identify significant differences. One-way ANOVA was selected based on the fact that our data sets were paired and independent columns. In addition, as the comparison is based on a set of means, Sidak's was chosen with more power compared to the other available methods. 

No statistically significant differences were observed between the data set for viability % of all tested excipients obtained from the MTT and CV assays, regardless of the tested cell lines. Rankings of cytotoxicity responses to the investigated excipients based on either the MTT or the CV assay were the same except for Labrafil® and Transcutol® in HaCaT cells.

**Figure 3:**
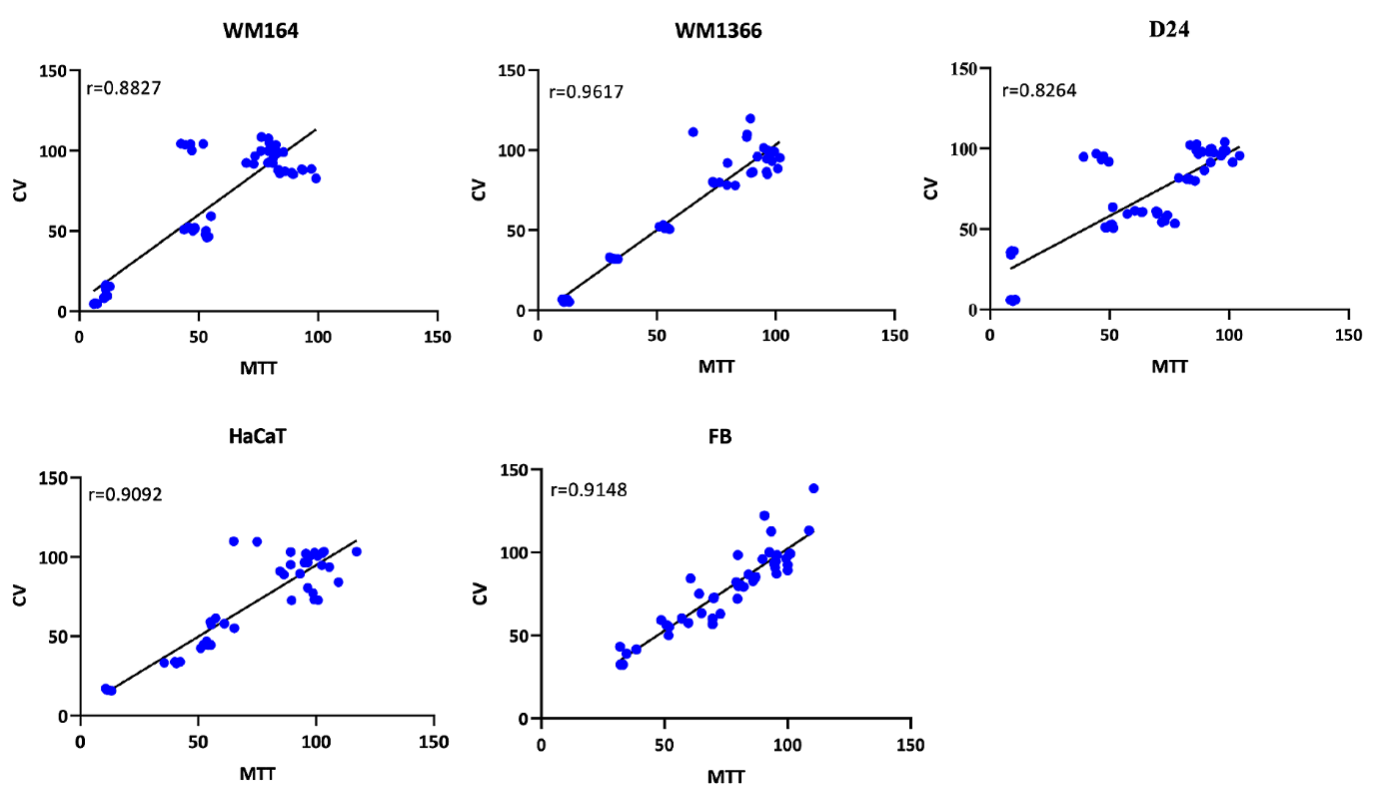
Correlation of cell viability values (%) between MTT and CV assays for all surfactants in each cell line. Each point corresponds to cell viability % value obtained after exposure to a surfactant. r= Pearson's correlation coefficient. ***p<0.001 denote significant correlation.

Figure 3(Fig. 3) indicates that regardless of the type of excipient, reasonable correlations (r>0.80) between cell viability determined by MTT and CV assays were observed for all excipients in every cell line (Figure 3(Fig. 3)).

**Figure 4:**
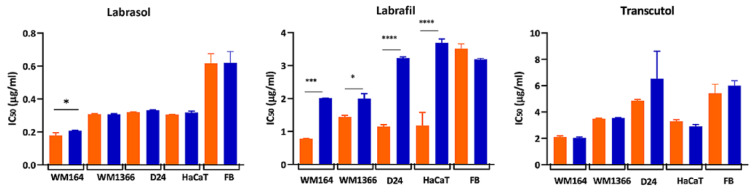
Cytotoxicity of the excipients expressed as IC_50_ (µg/ml) on the tested cell lines and measured by MTT and CV assays. Data are presented as mean ± SD of two independent experiments, performed in triplicate. MTT and CV assays were compared by the One-way ANOVA with Sidak's post hoc test (p values of **** <0.0001; *** <0.001; ** <0.01; * <0.5) denote significant differences.

To compare the sensitivity of the assay methods in detecting the toxicity level of the excipient, the estimated IC_50_ values for each individual excipient calculated from both assays were compared with each other in every cell line by ANOVA analysis for significant differences (Figure 4(Fig. 4)). Significantly lower MTT IC_50_ compared to CV was seen in all cell lines for Labrafil®, with the exception of fibroblast cells, and in WM164 cells for Labrasol®. In the rest of the cases, there were no significant differences between the two assays.

### Trypan blue exclusion assay 

**Figure 5:**
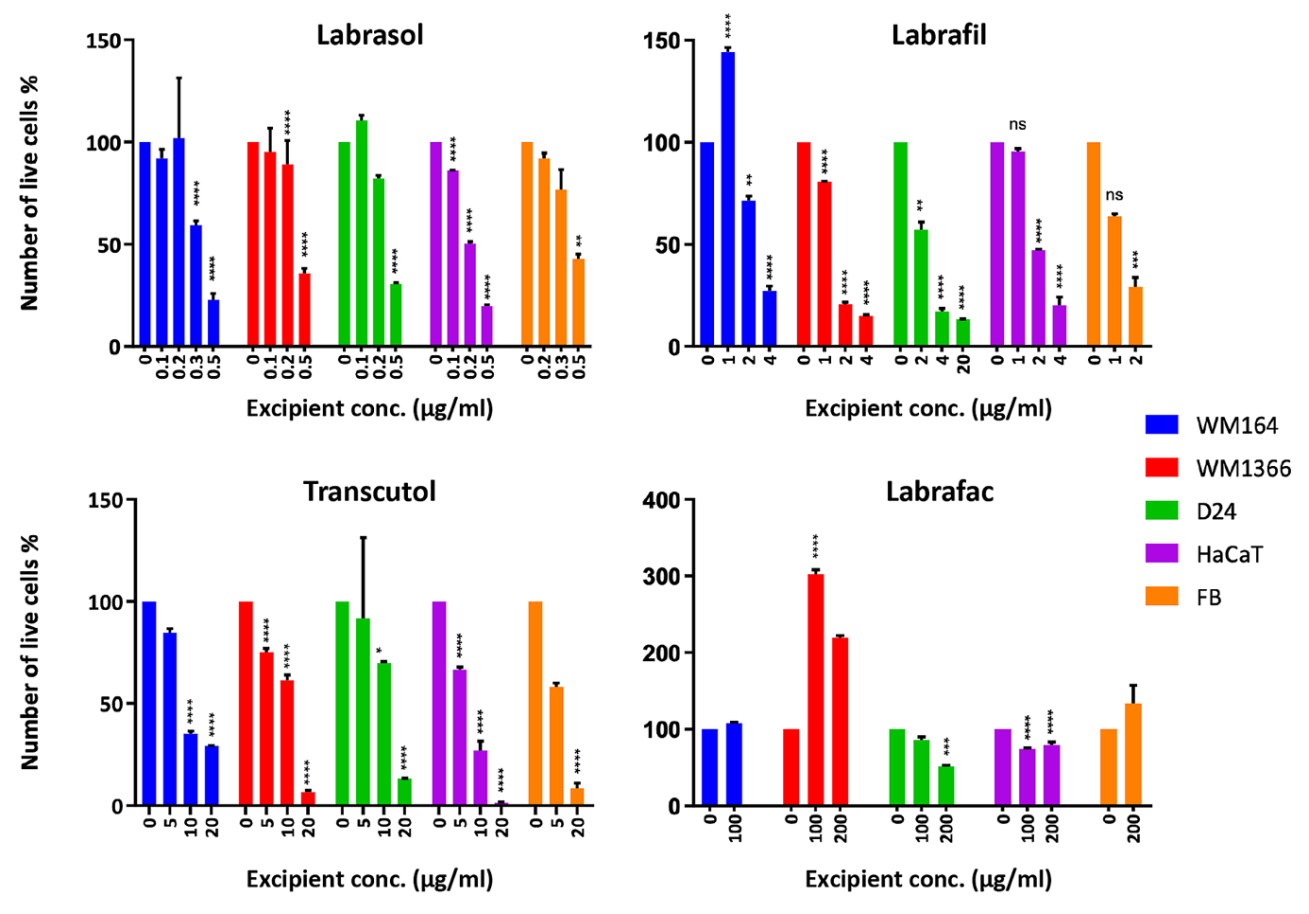
Cell viability % obtained from trypan blue exclusion assay for the excipients after 24 hr on the tested cell lines. Live cells are shown as a percentage of the untreated control cells. Data are presented as mean ± SD (n=3-5). Data were analyzed using One-way ANOVA. (p values of **** <0.0001; *** <0.001; ** <0.01; * <0.5) denote significant differences from the control value.

The trypan blue exclusion assay was performed to examine the integrity of the plasma membrane after exposure to the excipients. Results showed a progressive decrease in live cell numbers after treatment with increasing excipient concentrations (Figure 5(Fig. 5)).

The IC_50_ values determined by TB assays for each excipient were compared with those from MTT and CV. Data are summarized in Table 4(Tab. 4). Due to the small group size, the non-parametric Kruskal-Wallis test was selected to analyse this data set. Analysis (p<0.05) showed that only the IC_50_ values for Transcutol® were significantly greater than the ones obtained from the two viability assays in all cell lines.

### Reactive Oxygen Species (ROS) levels

Generation of ROS is known to be one of the downstream impacts of oxidative stress causing mitochondrial dysfunction (Adam-Vizi and Chinopoulos, 2006[3]; Brookes, 2005[15]). Hydrogen peroxide is known to increase the level of ROS in many cell types (Masaki and Sakurai, 1997[56]) and in this study it was used as a positive control for cellular ROS generation. 

Dihydrorhodamine 123 (DHR) is an oxidation-sensitive fluorometric dye that penetrates cells and localizes into the mitochondria through its lipophilic properties. If present, mitochondrial ROS could oxidize DHR and convert it to an extremely stable fluorescent product, rhodamine-123 (R123) (Villamena, 2017[91]). DHR has been commonly used to detect ROS levels in cellular models (Ranganathan et al., 2009[73]). To investigate possible cell death mechanisms, we applied DHR and compared ROS generation in response to pharmaceutical excipients, compared to a peroxide control.

Cells were exposed to various concentrations based on the cytotoxic potency of each excipient. The range of applied concentrations was selected to cover upstream and downstream of the IC_50_ concentrations calculated from both viability assays, as shown in Table 3(Tab. 3). For Labrasol®, cells were treated with concentrations of 0.1, 0.3, 0.5 and 1 µg/ml. For Labrafil® concentrations of 0.2, 2, 5 and 10 µg/ml were used. Transcutol® was applied in concentrations of 5, 10, 20 and 50 µg/ml.

**Figure 6:**
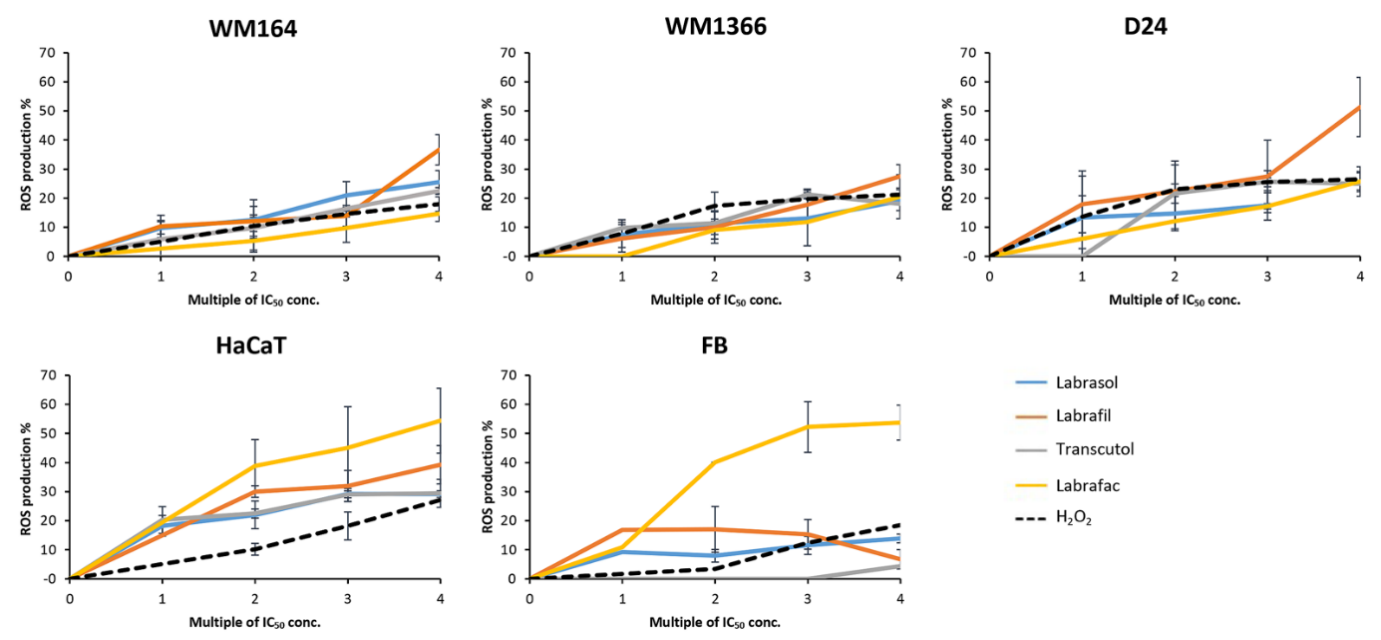
ROS intensity after cell exposure to increasing concentrations of excipients. Excipient concentrations are plotted from 0 to high on X axis (as the applied concentrations are varied depending on their toxicity, the applied doses are referred to as (0 to some multiple of IC_50_ concentration relative to the cell line and the tested excipient and ROS generation % on Y axis (ROS production results calculated as percentage of untreated control). Data are expressed as mean ± SD of 3 replicates.

The cells treated with the tested excipients showed an increase in ROS intensity in a dose-dependent manner compared to untreated control cells (Figure 6(Fig. 6)).

In this section, to avoid repeating the name of the cell line for the ROS production level, cell lines are categorized as two groups of cell type; melanoma cells and normal cells. Below, the melanoma cell lines are referred to in the order; WM164, WM1366, D24 and normal cells in the order; HaCaT, FB.

The results in Figure 6(Fig. 6) show that the greatest applied concentration of Labrasol® stimulates ROS production by 25.5 %, 19.04 %, and 20.9 % respectively in melanoma cells and by 29.4 % and 13.89 % in normal cells respectively. 

Labrafil® induced ROS generation by 36.63 %, 27.57 %, and 51.35 % respectively in melanoma cells and 39.23 %, and 6.7 % in normal cells, respectively.

Transcutol® increased ROS production by 22.5 %, 18.1 %, and 24.9 % respectively in melanoma cells and by 29.4 %, and 4.3 % respectively in normal cells.

The results of quantitative measurement of ROS levels indicated that highest applied concentration of Labrafil® stimulated the greatest level of ROS generation in all melanoma cells. HaCaT cells were more sensitive than the other tested cells towards ROS production while normal fibroblasts showed minimal sensitivity towards ROS production after excipient treatments.

**Figure 7:**
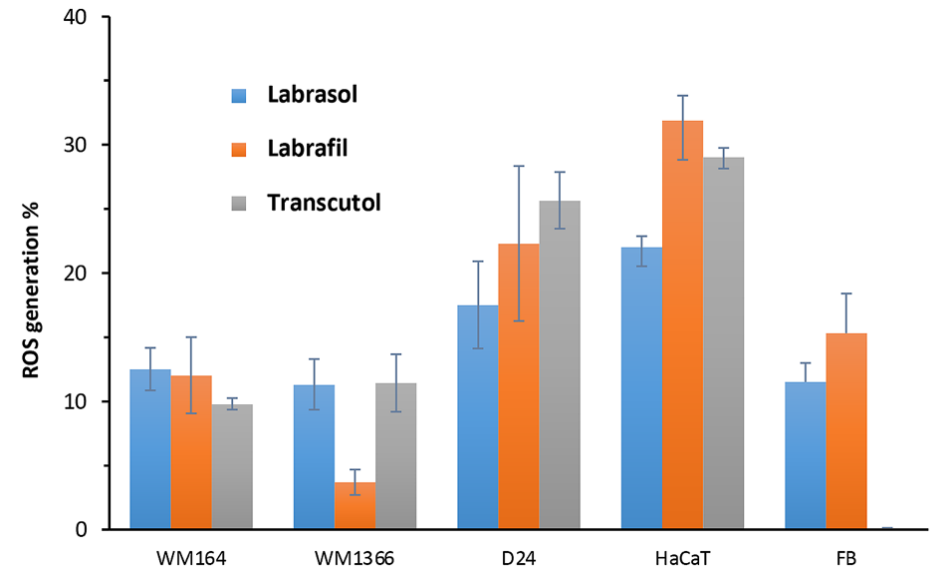
ROS generation in each tested cell line after 24 hr exposure to the excipients at their IC_50_ concentration. ROS production results are calculated as percentage of untreated control. Data are expressed as mean ± SD of 3 replicates.

Figure 7(Fig. 7) shows the percentages of ROS generation at IC_50_ concentrations in each cell line for the tested excipients. Among the cancerous cells, the highest percentage of ROS production occurred in the non-mutated melanoma cell line (D24) at exposure to all tested excipients. In normal cells, HaCaT shows more production of ROS after contact with all tested excipients.

### Cell cycle analysis

Flow cytometry is capable of differentiating specific subsets of cells within a mixed cell population. Analyzing the kinetics of the cell cycle in combination with the cell responses to treatment could provide a comprehensive picture of the relationship between cell cycle events and apoptosis in a cell cycle perspective (Sherwood and Schimke, 1995[83]). Any damage to DNA could interrupt the cell cycle progress and arrest the cells at certain checkpoints. This arrest can facilitate DNA repair to avoid carcinogenesis or lead to cell death, normally by apoptosis. Understanding the chemical influences of these processes not only provides new therapeutic approaches and tools to enhance the killing efficacy of major cancer therapeutics but also directly increase cancer cell death (Visconti et al., 2016[92]). 

The effectiveness of the excipients on the cell cycle progress and in inducing apoptosis was assessed by flow cytometry and PI staining. The cell cycle profile results of the untreated cells for G_0_G_1_, S and G_2_M phases show 48.5 %,35.7 %, and 10.4 % for WM164; 36.4 %,13.2 %, The effectiveness of the excipients on the cell cycle progress and in inducing apoptosis was assessed by flow cytometry and PI staining. The cell cycle profile results of the untreated cells for G_0_G_1_, S and G_2_M phases show 48.5 %,35.7 %, and 10.4 % for WM164; 36.4 %,13.2 %, and 32.6 % for WM1366; 36.4 %, 46.2 %, and 16.1 % for D24; 56.3 %, 30.2 %, and 10.3 % for HaCaT; and 39.5 %, 52.2 %, and 13.5 % for primary Fibroblast cells respectively (Table 5(Tab. 5)).

**Figure 8:**
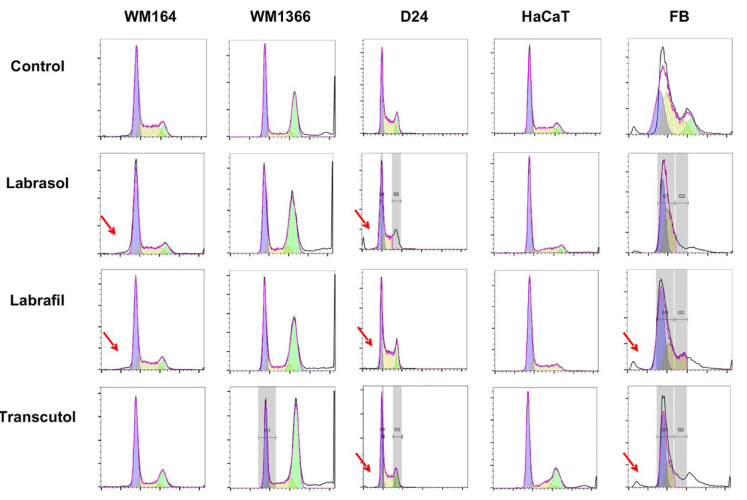
The effects of excipients on the process of the cell cycle of human melanoma and non-melanoma cells. The FACS diagram of the cell cycle. PE-CY5 channel indicates the fluorescent intensity of PI, and the Y axis indicates cell number (events). Red arrow shows the apoptotic population.

To assess the effect of treatment in cell cycle progress, we compared the DNA histogram between control and sample groups treated with IC_50_ concentrations (Figure 8(Fig. 8)).

Labrasol® only increases the cell population in sub G_0_G_1_ in melanoma WM164 and D24 cell lines, indicating increases in early apoptotic population in these cell lines. It does not influence the WM1366 cell line or normal HaCaT or Fibroblasts.

Labrasol® also showed effects on the G_0_G_1_ phase in WM164 cells and increased the cell population percentage which was accompanied by a proportional decrease in the percentage of cells in the S and G_2_M phase. However, Labrasol® interrupted the cell cycle of WM1366 by increasing the cell population in G_2_M phase with a concurrent reduction in the cell population in the G_0_G_1_ phase. In the D24 cell line, Labrasol® increased populations in both the G_0_G_1_ and G_2_M phases, with a corresponding decrease in the S phase of cellular progression. In normal HaCaT cells after treatment with Labrasol®, the population in G_0_G_1_ increased, while decreases were seen in the S and G_2_M phases. In normal fibroblasts, Labrasol® caused cell accumulation in the G_0_G_1_ and S phases and completely inhibited cell progression into the G_2_M phase.

The interaction of Labrafil® with the WM164 cell cycle was similar to Labrasol®, increasing sub G_0_G_1_ populations of early apoptotic cells and in G_0_G_1_, with a corresponding decrease in the S phase. The effects of Labrafil® in WM1366 were also similar to Labrasol®, with increased cellular populations in the G_2_M phase and reductions in G_0_G_1_. In normal cells, Labrafil® completely inhibited cells entering the G_2_M phase, while causing accumulation in the G_0_G_1 _and S phases. However, in normal Fibroblasts it caused a small increase in the sub G_0_G_1_ cell population.

Transcutol® disrupted cell cycle progression by increasing the cell population in sub G_0_G_1_ phase of WM164, D24 melanoma cells, and normal Fibroblasts. In the WM164 cell line, it increased cellular accumulation in both G_0_G_1_ and G_2_M checkpoints with a corresponding decrease in the S phase. In WM1366, it had little effect except for a slight rise in the G_2_M phase population. In D24 cells, Transcutol® caused an increase in the G_0_G_1_ population, with related reductions in S and G_2_M phases. In normal HaCaT cells, Transcutol® increased the population in G_2_M phase, while in normal Fibroblasts, it completely inhibited the G_2_M phase and increased both the G_0_G_1_ and S phases.

The overall results indicate that all three tested excipients; Labrasol®, Labrafil® and Transcutol® affected cell cycle progress in WM164 and D24 melanoma cells. In these two cancerous cell lines, the IC_50_ concentrations of Labrasol® and Labrafil® could induce apoptosis followed by arresting the melanoma cells at the G_0_G_1_ and G_2_M checkpoints. In WM1366 cells, the IC_50_ concentration of the excipients was insufficient to induce apoptosis and cells could only be arrested at the G_2_M checkpoint. After exposure to the excipients at their IC_50_ concentrations, HaCaT cells did not progress to the apoptotic phase and were arrested in the G_0_G_1_ and S phases. On the other hand, low populations of apoptotic cells were generated in the normal FB cell line. In summary, we concluded that the excipients had inhibitory effects on cell cycle progress at various levels.

See also the Supplementary data.

## Discussion

Many topical products are reliant on the penetration enhancing capacities of excipients to allow the active drug to overcome the stratum corneum barrier and reach its target. This may come at the cost of increased toxicity to the underlying layers of viable cells. A good understanding of excipient toxicity and the linked mechanisms can provide valuable tools for formulators. This can be of particular importance in the generic industry where replacement of excipients in a “like for like” fashion may be required in the absence of excipients that are no longer available. Where the overall performance in terms of the penetration is required to be within the same range of acceptability as the reference product, changes in the level of toxicity resulting from the excipient replacement is also considered. According to the FDA Inactive Ingredient Guide (IIG) (FDA, 2019[30]), during generic product development, changes of the reference formula in terms of excipient replacement, grade of excipient, or quantity of excipient incorporated in the formula, etc. need to be justified by its functionality, pharmacology/toxicology data, and bioequivalence/clinical data. However, due to the lack of direct regulation, cost and patenting issues, a generic firm may have to reformulate their product without performing a comprehensive evaluation of the new ingredients' toxicity behavior (Chang et al., 2013[18]).

In vitro cell culture is a well-established model that has been used for cell toxicity evaluation and prediction of the skin irritancy potential of chemical substances (Mullerdecker et al., 1994[60]). Previous studies showed that application of a battery of *in vitro* assays could be a reliable pre-clinical approach to predict toxicity of chemical in various cell types (Nogueira et al., 2011[62]). A combination of MTT and crystal violet assays has been used previously to analyze the effects of various drugs in cancer cells (Klingenberg et al., 2014[46]). 

Previous studies have demonstrated that the results of cell-based toxicity assays are impacted by the solvent and procedural steps applied to introduce the test chemical to the cells. Some physicochemical properties of the test chemical combinations such as lipophilicity and volatility as well as procedural steps in solution preparation can lead to inhomogeneous distribution of the chemical, resulting in significant quantitative differences in toxicity. For cell treatment, indirect dosing, or dosing the cells with a previously prepared mixture of the exposure medium and the stock solution, is recommended (Tanneberger et al., 2010[87]). Therefore, careful attention was paid in this work in preparing treatment solutions to avoid any technical errors. 

Here we studied the cytotoxicity of some topical excipients with potential interest in the pharmaceutical industry as well as sensitivity of several cancerous and non-cancerous cell lines to the toxic effects of the tested excipients. Comparison between the results of cell viability % obtained by the two different assays (MTT and crystal violet) showed no significant differences at 0.05 % (p<0.05) (ANOVA with Sidak's post hoc test). In addition, a good level of correlation was obtained between the test results. Based on our overall results, these assays could be considered suitable for the evaluation of cytotoxicity.

However, some exceptions were noted. The IC_50_ results from Labrafil® show that the MTT assay was more sensitive in detecting cell damage than CV staining, regardless of the cell type. Furthermore, in the case of Labrasol®, the IC_50 _result obtained in WM164 with MTT was significantly lower than that obtained with CV. These differences between assays are not unanticipated and have been reported previously as the cellular response varied based on the mechanism of cytotoxicity assay in revealing loss of viability (Burlando et al., 2008[17]; Schröterová et al., 2009[79]). The MTT assay is associated with metabolic activity of live cells (Berridge et al., 2005[13]), while the CV assay is insensitive to alterations in cellular metabolism and consequently, it is not reflective of the metabolic changes that may be initiated by toxins. The CV assay is appropriate for the assessment of the impact of compounds on cell survival and growth inhibition (Almutary and Sanderson, 2016[4]; Feoktistova et al., 2011[31]). This suggests that the mechanism of toxicity caused by these surfactants involves an early impact on the metabolic activity of the cells, while cell lysis could be affected at a later stage. Toxin exposed cells could become metabolically deactivated before proceeding to a state of complete death and detachment from the well. In this condition the total DNA mass present could be greater than that contributed by just the metabolically active cells in each well.

We found no significant differences between the viability data recorded from MTT and CV for Labrafil® and Labrasol® in all cell lines, with good correlations of these two assays for all excipients in each cell line. This good relationship between the two endpoints even when individual differences were observed within experimental data agrees with the literature, where the nature of the assay and different toxicity influences from the surfactants have been recognized. Based on our results, we recommend that the inter-correlation data from the two assays be carefully evaluated before they can be regarded as interchangeable for the assessment of cytotoxicity. The combination of several endpoints with different mechanistic aspects might be useful in order to differentiate between the effects on cellular activity or overall cell death.

The cell viability results obtained from the MTT and CV assays generally correlated well with those from the trypan blue exclusion assay. However, in the case of Transcutol®, the IC_50_ values obtained from the trypan blue assay in all cell lines were significantly higher than those from either of the viability assays. Transcutol® has been shown to be preferentially incorporated into more polar regions of multiple lipid bilayers, resulting in an increase in the spaces between lipids without a significant modification of the bilayer structure (Osborne and Musakhanian, 2018[63]). In this way, the general integrity of the membrane can be maintained, while creating only enough space for small molecules like Transcutol® (134.17 g/mol) to pass, while larger molecules such as trypan blue (960.8 g/mol) may be trapped within the membrane or completely excluded. Therefore, the dead cells resulting from Transcutol® incorporation may appear unstained and be counted as live cells. Results from this work could be explained by the fact that Transcutol® may make the cell membrane selectively permeable by excluding larger molecules like trypan blue and resulting in false negative staining in this cytotoxicity assay.

Triton X-100, a non-ionic surfactant that is a membrane-lytic and highly cytotoxic, is commonly used as a toxic reference compound in cell viability assays. The IC_50_ value of Triton-X 100 measured by the MTT assay in human leukemic cells (CCRF) was previously reported to be 1 µg/ml (Duncan et al., 1994[25]), much greater than that found in this study (<0.08 µg/ml). However, it is well known that the cytotoxic effects of chemical compounds depend on the tested cell type (Bačkorová et al., 2011[7]; Schröterová et al., 2009[79]). Duncan and colleagues (1994[25]) studied the cell viability in different cell type which could explain this variation between our result and those reported in a different cell line.

**Figure 9:**
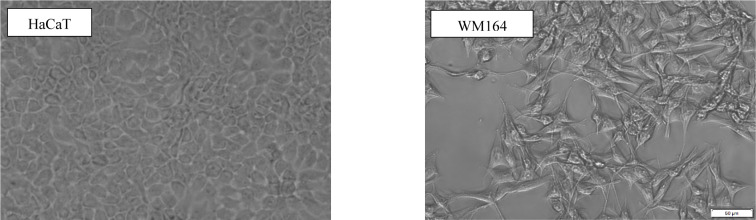
Bright field microscopy of HaCaT and WM164 at confluence. The different morphology and cell growth arrangement due to the contact inhibition (HaCaT) and loss of this property (WM164). Scale bar=50 µm.

Of the three cancer cell lines used in this work, WM164 was the most sensitive to the toxic effects of the tested excipients. Distinct sensitivity of cell lines against a chemical compound could be partially explained by the features of each cell type which lead to a different defence mechanism against toxic exposure. Contact inhibition is a fundamental property of cells and loss of this feature is associated with malignancy and tumorigenesis (Morais et al., 2017[57]). The effects of contact inhibition, or its lack, on cell proliferation can be observed in culture-based experiments. Contact inhibition can cause a decrease in proliferation rates at high cell density (Abercrombie, 1970[1]). In the present study, the cancerous WM164 cells in culture showed a lack of contact inhibition by growing in spatial layers and forming clusters (Figure 9(Fig. 9)). Uncontrolled proliferation leading to high differentiation rates may explain the increased sensitivity of WM164 towards the toxic effects of the excipients. In contrast, HaCaT and primary FB cells showed greater resistance to the toxicity of the excipients. A study on cytotoxicity and phototoxicity showed HaCaT keratinocytes to be more resistant than other human skin derived cell lines due to their lower growth rate, resulting from contact inhibition which controls the rate of proliferation and metabolic processes of the cells (Sha et al., 2005[82]). Contact inhibition is also regarded as a characteristic feature of fibroblast-like cells in culture (Abercrombie, 1970[1]). In our experiments, WM1366 showed very similar morphological and phenotypic characteristics to the HaCaT cell line. These two cell lines had similar growth rates and contact inhibition properties which could partly explain their similar responses towards toxicity.

Our results demonstrated a greater toxicity for Labrasol® in all cell lines, with IC_50_ values ranging from <0.2-0.6 µg/ml, and a low toxicity for Transcutol® (IC_50_ 2-5.5 µg/ml). Previous studies investigated the cytotoxicity of Labrasol® and Transcutol® in various cell lines, including human cervical cancer (HeLa) and human epithelial colorectal adenocarcinoma (Caco-2) cells, by measuring MTT endpoints (Ujhelyi et al., 2012[89], 2015[90]). The results showed high toxicity for Labrasol® with IC_50_ values of approximately 2 µg/ml and lower toxicity with Transcutol®, with an IC_50_ of 34 µg/ml in the HeLa cell line. CaCo-2 cells also had similar cytotoxic response results to Labrasol® with 2 µg/ml IC_50_. The variation in IC_50_ is expected between different cell lines due to different sensitivity of the cells but the order in cytotoxicity ranking of the Labrasol® and Transcutol® is consistent with the high toxicity of Labrasol® and low toxicity for Transcutol® in our tested cell lines.

Critical micelle concentration (CMC), the surfactant concentration at which micelle formation occurs, is a property that is used to characterize surfactants (Perinelli et al., 2020[70]). CMC describes the arrangement of surfactants into micelles, which has an indirect effect on solubilization of lipophilic molecules including cellular membrane lipids (Korhonen et al., 2004[47]). CMC has been reported to be a useful parameter to characterize surfactant toxicity and has been recommended to be used as a reference concentration when comparing the toxic effects of surfactants of homologous groups (Inácio et al., 2011[41]) and the comparison between IC_50_ values and CMC values have been performed previously (Lucarini et al., 2018[54]).

Hydrophilic surfactants such as Labrasol® have low CMC with good solubilizing ability. They also have the ability to increase membrane lipid solubility, a non-selective mechanism that may contribute their toxic effects during cellular interaction (Ujhelyi et al., 2012[89]).

The cytotoxicity of Labrasol® has been investigated in Caco-2 cells, where the IC_50_ was reported to be 2 µg/ml, well above its CMC of 0.12 µg/ml (Ujhelyi et al., 2012[89]). In contrast, we showed that Labrasol® expressed toxicity in our tested cell lines far below the CMC, with IC_50_ values in the range 0.02 - 0.05 µg/ml. In another study, the relationship between toxicity and structure of surfactants as well as target cell type in fully polarized and confluent epithelial cells, confluent but non-polarized epithelial-like cells, dendritic cells, and human sperm was investigated. The authors reported toxicity in all cell types to some surfactants, including Triton X-100, monolaurin, DDPS and SDS at concentrations around their CMC, indicating a non-selective mechanism in their cell membrane destabilization. On the other hand, all tested cationic surfactants showed toxicity at concentrations far below their CMC, with significant differences in their toxicity towards different cell types. In these cases, in addition to cell membrane destruction, an intracellular mechanism characterized by membrane partitioning (Abreu et al., 2004[2]) and/or translocation across the membranes (Moreno et al., 2006[58]) was suggested. 

Therefore, the lower cytotoxicity of Labrasol® compared to CMC in our cell lines could be explained by involvement of modes of action other than membrane disruption. This action could be induced by Labrasol® at an intracellular level before any membrane disruption that causes cytotoxicity at very low concentrations. However further investigation is required for verification.

This work also examined the mechanisms of cell death caused by excipients applied to skin cell lines. Apoptosis or programmed cell death is known to be a highly regulated physiological mechanism to eliminate abnormal cells and its screening remains a gold standard method in cell death investigations for toxicity and anti-cancer drug discovery (Inácio et al., 2011[41]). In many cancer cells, generation of ROS during the apoptosis process as a response to toxicity leads to permeabilization of the mitochondrial membrane and release of pro-apoptotic factors (Sathiyamoorthy and Sudhakar, 2018[78]). However, ROS functions as a double-edged sword, causing benefit or harm to the cells depending on the level of production and cell type. Low to moderate ROS levels are vital for survival of normal cells, including proliferation and metabolism, while excessive levels of ROS can kill the cells. On the other hand, in cancerous cells, increased ROS production can lead to persistent tumor cell survival by ROS adaptation leading to resistant cells (Perillo et al., 2020[69]). 

To understand whether ROS generation in our cells was involved in increased cell death, cell cycle and pre-apoptotic analysis was performed. We found that exposure to the tested excipients in all cell lines caused elevated ROS levels compared to untreated control cells, but this was not always associated with increased populations of apoptotic cells. Previous studies have reported cytotoxicity of surfactants that led to cellular arrest and apoptosis via ROS mediated pathways (Borner et al., 1994[14]; Zorov et al., 2014[95]). However, in this study, ROS influence on the treated cells was primarily through cellular arrest in DNA checkpoints and secondarily, by pushing low percentages of the cells into the apoptosis phase. We concluded that the cell repair system was able to manage the DNA related damage resulting from treatment with IC_50_ concentrations of these excipients without causing significant apoptosis. However, the increased ROS levels may have contributed to cellular vital mechanisms and/or to other types of cell death such as autophagy and necroptosis (Han et al., 2013[40]). This requires further investigation.

*In vivo* responses of excipient mixtures have previously been reported in a number of studies, notably by Maibach and others, for instance (Kartono and Maibach, 2006[44]; Rhein et al., 1990[74]). Synergistic responses to excipient mixtures were observed in the work of Kartono and Maibach. *In vitro* testing of combined excipients has also been reported but tends to be limited to specific endpoints, particularly the use of a single cell line for viability testing (Soltani et al., 2022[84]). In this work, we chose to expose cells to single excipients only, rather than mixtures. We used several different techniques: (i) skin toxicity by two different cell viability assays (MTT and crystal violet), on 5 different cell lines (normal human keratinocytes, primary human skin fibroblasts and three cancerous human cell lines), (ii) cell membrane integrity using trypan blue, (iii) generation of reactive oxygen species (ROS) using dihydrorhodamine, and (iv) cell cycle analysis by flow cytometry, to reveal the importance of apoptotic processes. In doing so, our aim was to establish the framework by which these techniques can be coordinated to obtain a clear picture of the responses to excipients.

Our work is significant because it seeks to evaluate the excipient responses both quantitatively and mechanistically. In the next phase of this work we can examine binary or other mixtures of excipients that are representative of in-use topical formulations.

However, while the *in vitro* assessment of cell viability as a measure of skin irritation potential of chemicals has long been a validated gold standard procedure, its relationship to the *in vivo* state is indirect. Unlike *in vivo*, in which deeper viable skin layers are protected by a skin barrier composed of non-viable cells in the stratum corneum, *in vitro* cells are directly exposed to chemicals. Consequently, for some purposes, skin models such as commercial human skin equivalents (Liu et al., 2020[53]) or human skin explants (Patrick et al., 2021[68]) with histological analysis are used to assess skin irritancy under what may be regarded as more in-use conditions. However, the OECD guidelines recommend assays using artificially reconstructed skin. These have poor barrier properties and are very expensive. Beyond these experimental methods, an increasing number of commercial and public domain *in silico* models is available for predicting skin irritation and sensitivity (Selvestrel et al., 2022[80]). Despite their limitations, the cell-based assays used here remain appropriate for use in screening or ranking of toxicants and for direct assessment of toxic mechanisms. A harmonized protocol in conjunction with robust statistics can be useful in establishing cell-based assays as an alternative.

## Conclusions

An *in vitro* system such as, for example, an immortalized cell line grown on a 2D surface, is a reliable pre-screening tool to rank toxicity levels of chemicals prior to performing further studies on more sophisticated models. This minimizes the need for animal tests. It can also be used to generate data for mechanistic interpretation, and in this work, we have included ROS generation studies and cell cycle analysis in a triangulation system to add the power of this approach. 

It is possible to draw some important conclusions from this work. First, the tested excipients produced different results for cell viability, ROS generation and cell cycle progression, indicating that they may activate different mechanisms, or mechanisms to different extents. This supports the vast majority of literature in this field where toxicant physicochemical properties are of major significance. Second, although there was some overlap in the MTT and CV assay results, it is clear that the *in vitro* assessment of cytotoxicity can be assay-dependent. This can assist with mechanistic understanding; for example, in this work, results from the trypan blue assay with Transcutol® allowed us to understand effects of Transcutol® on cell membrane permeability. Assay dependence also highlights the need for standardized end point assays. Third, normal and melanoma cell lines showed different responses to the test excipients. Our results from the range of viability, ROS and cell cycle tests provided an insight into cytotoxicity mechanisms that going forward, could assist in developing improved cytotoxic cancer therapies. The tested excipients predominantly caused cell cycle arrest in the checkpoints to facilitate DNA repair prior to mitosis but did not induce apoptosis. Investigations into the generation of reactive oxygen species (ROS) revealed that exposure to the tested excipients caused elevated ROS levels in all cell lines that could be associated with cellular essential metabolic activity and/or to other types of cell death such as autophagy or necrosis. The greater sensitivity to excipient exposure shown by the WM164 cells can be explained by their relative lack of cell contact inhibition compared to the normal and other cancer cells.

As noted in the Introduction, it is unlikely that a single mechanism of cytotoxicity applies in all cases. Mechanisms have been widely explored in *in vitro* experiments with specific toxicants, using various model systems or cell lines and end point assays. More work is required to define the various parameters such as toxicant physicochemical properties that generate specific mechanistic pathways, as well as the most appropriate skin models or cell lines that should be used. A standardized set of end point assays is also required. Further work to elaborate the mechanisms of cytotoxicity induced by tested excipients can be done by applying apoptotic/necrotic assays to distinguish different pathways to cell death within the exposed cell population. This would be helpful in understanding the mode of action of the excipients in inducing toxicity and their potential *in vivo* irritancy. 

This work has concentrated on developing a coordinated set of experiments to examine skin toxicity in common pharmaceutical excipients. Further work is required to use this approach by expanding it to deal with more in-use scenarios. In addition, the following topics require investigation.

Formulations applied to the skin contain one or more active compounds and a range of excipients, and as shown in *in vivo* and some *in vitro* studies, synergistic responses may occur by the interaction of more than one chemical. Our study design can be used to investigate this. 

Also discussed above, the way in which they are applied, often to exposed body sites, means that skin products are subjected to a variety of degradative processes initiated by heat, light, etc. It is important to consider the toxicity of not only the pure actives and excipients, but also the degradation products, some of which may become toxic, or increase in toxicity, compared to the pure compound.

Finally, analogous to predictive models of *in vivo* skin permeation based on *in vitro* data, further work is needed to use *in vitro* skin toxicity data to predict *in vivo* responses, and to assess the correlations between *in vitro* and *in vivo* data with *in silico* predictions.

## Notes

Yousuf Mohammed and Jeffrey E. Grice (Therapeutics Research Group, Frazer Institute, The University of Queensland, Woolloongabba, QLD 4102, Australia; Tel.: +61-7-344-38032, E-mail: jeff.grice@uq.edu.au) contributed equally as corresponding author.

## Declaration

### Author contributions

Conceptualization, YM and JEG; formal analysis, FF, HSN and JEG; investigation, FF; methodology, FF and YM; resources, MSR; supervision, JEG; writing - original draft, FF; writing - review & editing, FF, YM, MSR and JEG. All authors have read and agreed to the published version of the manuscript.

### Acknowledgments

This research was carried out at the Translational Research Institute, Woolloongabba, QLD 4102, Australia. The Translational Research Institute is supported by a grant from the Australian Government. MSR was supported by an Australian National Health & Medical Research Council Senior Principal Research Fellowship (1107356). Human melanoma cell lines were kindly provided by Associate Professor Helmut Schaider's laboratory (University of Queensland, Frazer Institute). The authors thank Dr Sarika Namjoshi for her comments in the planning and discussions.

### Funding

This research received no external funding.

### Institutional review board statement

Not applicable.

### Informed consent statement

Not applicable. 

### Data availability statement

The data presented in this study are available on request from the corresponding author.

### Conflicts of interest

The authors declare that they have no conflict of interest.

## Supplementary Material

Supplementary data

## Figures and Tables

**Table 1:**
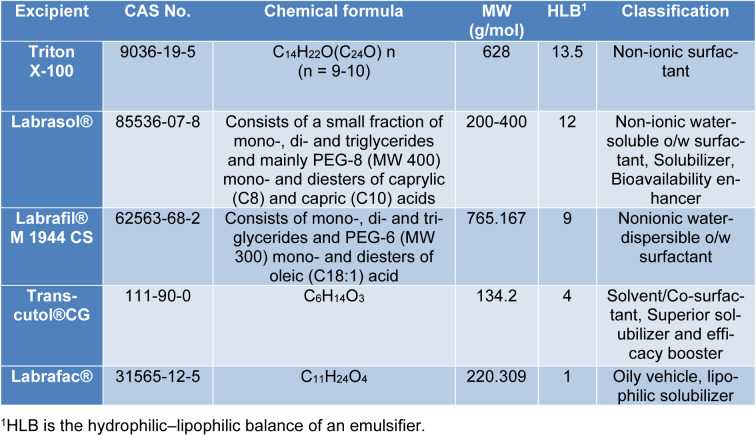
List of tested excipients

**Table 2:**
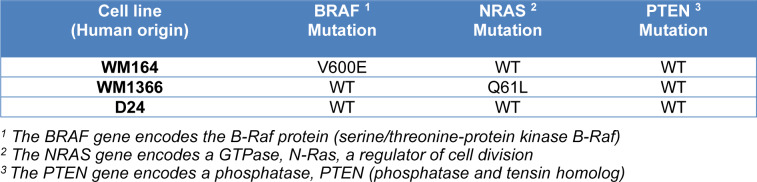
List of tested cancerous human cell lines. [WT (Wild Type) represents the non-mutated gene]

**Table 3:**
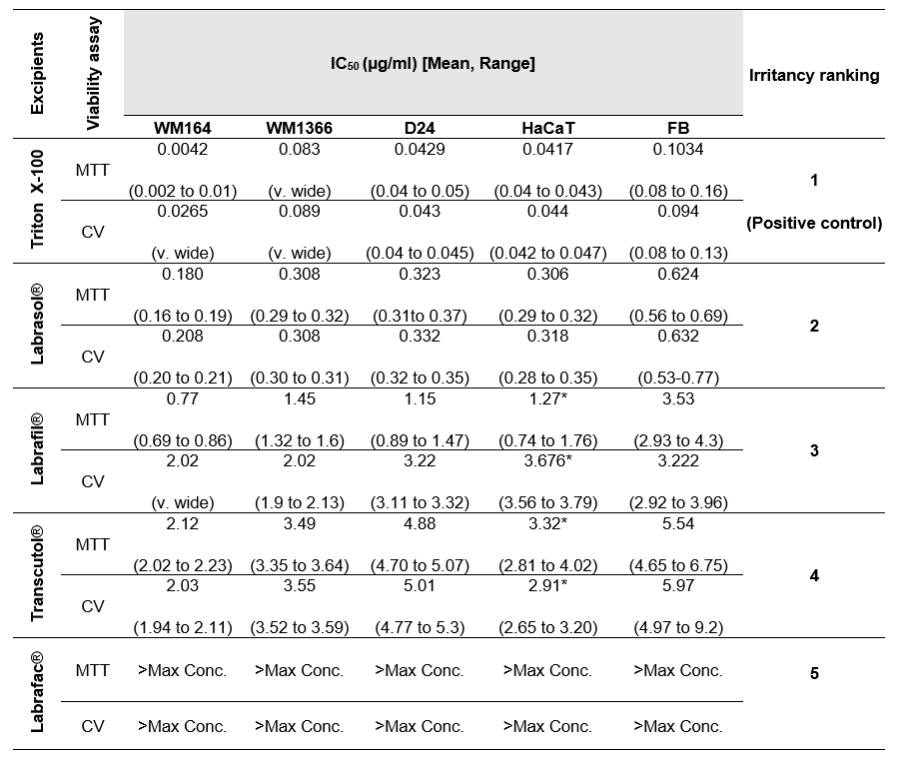
IC_50_ values (µg/ml) of tested excipients obtained by MTT, CV assays in the used human cell lines. R^2^>0.95. n=minimum of 5-6 replicates. [* The IC_50_ does not match the ranking.]

**Table 4:**
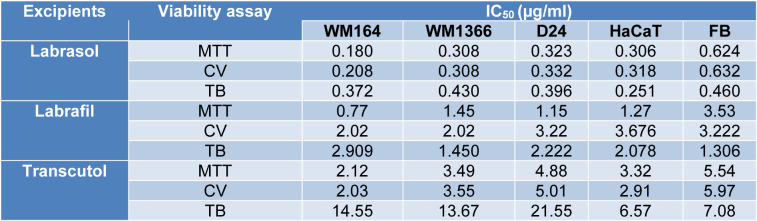
IC_50_ values obtained from trypan blue, MTT, and CV (µg/ml)

**Table 5:**
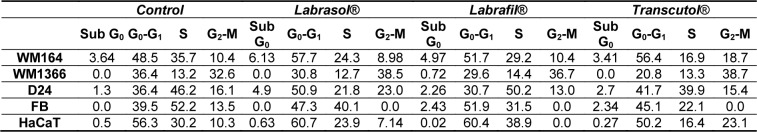
DNA distribution (%) during cell cycle after 24-h treatment with the tested excipients
