# Supplementary material for: In vitro screening of topical formulation excipients for epithelial toxicity in cancerous and non-cancerous cell lines
Source: EXCLI J. 2023 Nov 16;22:1173–99. doi: 10.17179/excli2023-6072 (PMC10776879; doi:10.17179/excli2023-6072)

EXCLI Journal 2023;22:1173-1199 – ISSN 1611-2156
Received: April 03, 2023, accepted: October 11, 2023, published: November 16, 2023

Supplementary data to:

Original article:

IN VITRO SCREENING OF TOPICAL FORMULATION EXCIPIENTS FOR EPITHELIAL TOXICITY IN CANCEROUS AND NON-CANCEROUS CELL LINES

Farzaneh Forouz[1,2], Yousuf Mohammed[1,2*], Hamid S. A. Shobeiri Nejad[3], Michael S. Roberts[1,4,5], Jeffrey E. Grice[1,*]

1 Therapeutics Research Group, Frazer Institute, The University of Queensland, Woolloongabba, QLD 4102, Australia
2 School of Pharmacy, The University of Queensland, Woolloongabba, QLD 4102, Australia
3 School of Mathematics, Physics and Computing, University of Southern Queensland, QLD 4350, Australia
4 School of Pharmacy and Medical Sciences, University of South Australia, Adelaide, SA 5000, Australia
5 Therapeutics Research Centre, Basil Hetzel Institute for Translational Medical Research, The Queen Elizabeth Hospital, Woodville, SA 5011, Adelaide, Australia

* **Corresponding authors:** Jeffrey E. Grice, Therapeutics Research Group, Frazer Institute, The University of Queensland, Woolloongabba, QLD 4102, Australia, Tel.: +61-7-344-38032; E-mail: jeff.grice@uq.edu.au
Yousuf Mohammed, Therapeutics Research Group, Frazer Institute, The University of Queensland, Woolloongabba, QLD 4102, Australia; E-mail: y.mohammed@uq.edu.au

https://dx.doi.org/10.17179/excli2023-6072

This is an Open Access article distributed under the terms of the Creative Commons Attribution License (http://creativecommons.org/licenses/by/4.0/).

Supplement 1
Raw data shows optical density (OD) measurement of dissolved purple formazan in DMSO observed from MTT assay **(S1-Tables 1-4)** and optical density (OD) measurement of dissolved crystal violet dye in DMSO from crystal violet (CV) assay **(S1-Tables 5-8)** after 24 h treatment. Different concentrations of four tested excipients Labrasol®, Labrafil®, Transcutol®, and Labrafac® were applied on three cancer cell lines (WM164, WM1366, and D24) and two normal cells (HaCaT and primary fibroblasts).

Raw data tables concerning:
Figure 1: Representative dose-response curves from 24-h exposure of the tested cell lines to the tested excipients Labrasol®, Labrafil®, Transcutol®, and Labrafac®
Figure 2: **a**) Cell viability % obtained from MTT assay and calculated as percentage of untreated control. **b**) Cytotoxicity of the excipients expressed as IC_{50} values (µg/ml) on melanoma (WM164, WM1366, and D24) and Normal (HaCaT and FB) cells and measured by MTT assay.
Table 3: IC_{50} values (µg/ml) of tested excipients and Triton-X-100 as positive control, obtained by MTT, CV assays in the used human cell lines.
Figure 3: Correlation of cell viability values (%) between MTT and CV assays for all surfactants in each cell line.
Figure 4: Cytotoxicity of the excipients expressed as IC_{50} (µg/ml) on the tested cell lines and measured by MTT and CV assays.

(-) These concentrations have not been tested in some cell lines. Excipient concentrations were selected based on the sensitivity of the tested cell lines.

EXCLI Journal 2023;22:1173-1199 – ISSN 1611-2156
Received: April 03, 2023, accepted: October 11, 2023, published: November 16, 2023

MTT assay (S1-Tables 1-4): After cell incubation for 24 h, the excipient-containing medium was removed, and 100 µl of MTT in PBS (5 mg/ml) diluted 1:10 in medium was then added in the dark. Plates were further incubated for 2-3 h, after which time the medium was removed and the purple formazan product was dissolved by adding 200 µl of DMSO to each well. Plates were then placed in a shaker for 5 min at room temperature and the absorbance of the resulting solutions was measured at 570 nm using a microplate reader (Multiskan™ FC Microplate Photometer).

S1-Table 1:

Cell line	WM164					WM1366					D24					HaCaT					Fibroblast				
**Labrasol (µg/mL)**																									
0.0	1.806	1.943	1.908	1.852	1.632	1.107	1.102	1.137	1.122	1.125	1.288	1.364	1.145	1.229	1.211	1.107	1.102	1.137	1.122	1.095	0.462	0.458	0.401	0.446	0.462
0.1	1.327	1.478	1.786	1.407	1.323	-	-	-	-	-	-	-	-	-	-	-	-	-	-	-	0.436	0.437	0.436	0.426	0.436
0.2	0.824	0.852	0.922	0.895	0.900	1.000	1.111	1.019	1.082	1.081	1.188	1.209	1.162	1.283	1.085	1.099	1.111	1.019	1.082	1.081	0.366	0.366	0.384	0.375	0.366
0.3	0.187	0.189	0.209	0.194	0.191	0.614	0.597	0.580	0.601	0.592	0.779	0.699	0.795	0.705	0.766	0.614	0.597	0.580	0.601	0.572	0.322	0.318	0.319	0.324	0.322
1	0.114	0.117	0.112	0.121	0.123	0.130	0.144	0.122	0.124	0.123	0.120	0.117	0.121	0.118	0.119	0.130	0.144	0.122	0.124	0.123	0.148	0.152	0.155	0.154	0.148

EXCLI Journal 2023;22:1173-1199 – ISSN 1611-2156
Received: April 03, 2023, accepted: October 11, 2023, published: November 16, 2023

S1-Table 2:

Cell line	WM164					WM1366					D24					HaCaT					Fibroblast				
**Labrafil (µg/mL)**																									
0.0	1.782	1.732	1.696	1.741	1.697	1.080	1.119	1.015	1.123	1.052	1.447	1.314	1.358	1.362	1.412	2.279	2.280	2.334	2.109	1.976	0.462	0.458	0.401	0.446	0.462
0.1	1.516	1.672	1.569	1.651	1.517	1.095	0.929	1.007	0.999	1.062	1.234	1.174	1.191	1.173	1.185	1.735	1.635	1.698	1.764	1.714	0.439	0.394	0.434	0.448	0.439
0.2	-	-	-	-	-	-	-	-	-	-	1.211	1.207	1.173	1.255	1.219	-	-	-	-	-	-	-	-	-	-
1	1.363	1.487	1.374	1.610	1.289	0.706	0.757	0.742	0.763	0.725	0.676	0.654	0.685	0.698	0.691	1.394	1.443	1.451	1.391	1.485	0.380	0.293	0.382	0.270	0.380
2	0.789	0.899	0.721	0.821	0.790	-	-	-	-	-	-	-	-	-	-	-	-	-	-	-	0.236	0.237	0.233	0.232	0.236
3	-	-	-	-	-	-	-	-	-	-	0.674	0.514	0.644	0.676	0.626	-	-	-	-	-	-	-	-	-	-
4	0.195	0.135	0.120	0.122	0.118	0.134	0.114	0.114	0.114	0.121	1.196	0.998	1.214	1.220	1.111	0.300	0.284	0.252	0.297	0.281	-	-	-	-	-
5	-	-	-	-	-	-	-	-	-	-	0.131	0.131	0.118	0.123	0.122	-	-	-	-	-	0.203	0.200	0.227	0.224	0.203
10	-	-	-	-	-	-	-	-	-	-	-	-	-	-	-	-	-	-	-	-	0.172	0.168	0.171	0.175	0.172

S1-Table 3:

Cell line	WM164					WM1366					D24					HaCaT					Fibroblast				
**Transcutol (µg/mL)**																									
0.0	1.782	1.732	1.696	1.741	1.697	1.080	1.119	1.015	1.123	1.052	1.4467	1.3142	1.3582	1.3615	1.4116	2.279	2.280	2.334	2.109	1.976	0.462	0.458	0.401	0.446	0.436
0.2	-	-	-	-	-	-	-	-	-	-	-	-	-	-	-	1.969	2.035	1.978	1.965	2.165	0.428	0.462	0.443	0.416	0.433
1	1.659	1.551	1.649	1.548	1.683	1.052	1.033	1.033	1.094	1.012	1.3547	1.2811	1.3262	1.3154	1.3082	1.307	1.258	1.294	1.287	1.290	0.415	0.398	0.398	0.446	0.436
2	0.847	0.919	0.896	0.929	0.919	0.859	0.828	0.843	0.827	0.802	1.1967	1.1261	1.1353	1.1206	1.1133	0.913	0.948	0.990	0.859	0.701	0.276	0.261	0.280	0.290	0.306
5	0.196	0.187	0.192	0.195	0.217	0.335	0.364	0.341	0.357	0.317	0.7255	0.6323	0.6996	0.6671	0.7209	-	-	-	-	-	0.285	0.253	0.229	0.251	0.296
10	-	-	-	-	-	-	-	-	-	-	-	-	-	-	-	-	-	-	-	-	0.143	0.142	0.142	0.141	0.140

S1-Table 4:

Cell line	WM164					WM1366					D24				HaCaT					Fibroblast				
**Labrafac (µg/mL)**																								
0.0	-	-	-	-	-	-	-	-	-	-	1.2875	1.364	1.1451	1.229	1.037	1.067	1.119	1.086	1.048	0.462	0.458	0.401	0.446	0.436
0.0	2.838	2.913	2.609	1.627	1.632	1.107	1.102	1.137	1.122	1.125	2.569	2.5531	2.5617	-	1.107	1.102	1.137	1.122	1.095	-	-	-	-	-
20	1.374	1.467	1.537	1.341	1.298	1.090	1.074	1.079	1.115	1.084	1.259	1.188	1.123	1.199	1.060	1.074	1.079	1.115	1.084	0.416	0.409	0.411	0.406	0.406
50	-	-	-	-	-	-	-	-	-	-	0.868	0.942	0.835	0.858	-	-	-	-	-	0.434	0.453	0.440	0.455	0.441
100	2.368	2.445	2.435	-	-	-	-	-	-	-	1.640	1.789	1.533	-	-	-	-	-	-	0.573	0.589	0.563	0.457	0.540
200	2.072	2.039	2.639	-	-	1.060	1.074	1.079	1.115	1.318	1.888	1.833	1.979	-	1.060	1.074	1.079	1.115	1.283	0.582	0.683	0.602	0.552	0.511
500	-	-	-	-	-	0.723	0.878	1.001	0.985	1.005	-	-	-	-	0.988	0.717	0.853	1.150	1.157	-	-	-	-	-

S1-Table 4: (Positive Control)

Cell line	WM164				WM1366					D24					HaCaT					Fibroblast			
**Triton X-100 (µg/mL)**																							
0.0	2.838	2.913	2.609	2.589	0.639	0.632	0.449	0.540	0.602	0.815	0.790	0.781	0.803	0.787	1.630	1.482	1.596	1.523	1.785	0.721	0.689	0.661	0.750
0.1	-	-	-	-	0.736	0.619	0.675	0.606	0.715	-	-	-	-	-	-	-	-	-	-	0.714	0.658	0.638	0.735
0.3	0.153	0.156	0.154	0.143	-	-	-	-	-	0.744	0.757	0.767	0.792	0.748	1.427	1.248	1.405	1.364	1.546	0.578	0.567	0.584	0.627
0.5	0.145	0.165	0.167	0.148	-	-	-	-	-	0.144	0.122	0.147	0.147	0.136	0.332	0.287	0.363	0.396	0.383	0.381	0.399	0.387	0.418
1	-	-	-	-	0.115	0.122	0.115	0.112	0.126	0.138	0.124	0.144	0.122	0.134	-	-	-	-	-	0.397	0.415	0.364	0.426
10	0.144	0.108	0.110	0.108	0.087	0.066	0.052	0.088	0.071	-	-	-	-	-	0.155	0.145	0.152	0.151	0.142	-	-	-	-

EXCLI Journal 2023;22:1173-1199 – ISSN 1611-2156
Received: April 03, 2023, accepted: October 11, 2023, published: November 16, 2023

Raw data shows optical density (OD) of dissolved crystal violet dye in DMSO at 570 nm (S1-Tables 5-8) after 24 h treatment. Different concentrations of four tested excipients Labrasol®, Labrafil®, Transcutol®, and Labrafac® were applied on three cancer cell lines (WM164, WM1366, and D24) and two normal cells (HaCaT and primary fibroblasts).

Cells were fixed by 4 % (v/v) paraformaldehyde in PBS for 10 minutes. The fixed cells were washed twice with PBS and covered with 250 µl crystal violet working solution. The plates were placed on a bench rocker with medium frequency (20 oscillations per minute) at room temperature for 20 minutes. The plates were emptied and washed under indirect stream of tap water for three times and inverted on filter paper. The plates were taped gently on the paper to remove any remaining liquid and air dried with no lid for minimum of 2 hr at room temperature. After the plates were photographed, blue dye was dissolved in DMSO and the optical density of each well was measured at 570 nm (OD570) with a plate reader (Multiskan™ FC Microplate Photometer).

S1-Table 5:

Cell line	WM164					WM1366					D24					HaCaT					Fibroblast		
**Labrasol (µg/mL)**																							
0.0	3.965	3.896	3.896	3.848	3.797	3.088	3.016	2.994	3.065	3.000	3.752	3.670	3.673	3.668	3.696	3.914	3.876	3.909	3.751	3.780	0.631	0.664	0.530
0.1	3.828	3.891	3.425	-	3.598	-	-	-	-	-	-	-	-	-	-	-	-	-	-	-	0.591	0.579	0.600
0.2	0.325	0.332	0.301	0.347	0.364	1.559	1.559	1.564	1.567	1.595	3.427	3.593	3.361	3.507	3.193	2.867	2.822	2.841	3.024	2.916	0.518	0.529	0.521
0.3	2.079	1.978	2.023	1.982	2.246	2.664	2.666	2.566	2.600	2.594	2.299	2.335	2.242	2.174	2.240	1.744	1.725	1.664	1.759	1.687	0.379	0.377	0.382
1	0.189	0.176	0.175	0.193	0.185	0.167	0.158	0.160	0.159	0.156	0.218	0.217	0.223	0.192	0.194	0.634	0.611	0.671	0.618	0.627	0.272	0.216	0.220

EXCLI Journal 2023;22:1173-1199 – ISSN 1611-2156
Received: April 03, 2023, accepted: October 11, 2023, published: November 16, 2023

S1-Table 6:

Cell line	WM164					WM1366					D24					HaCaT					Fibroblast		
**Labrafil (µg/mL)**																							
0.0	3.634	3.865	3.658	3.821	3.605	3.218	3.208	3.018	2.998	3.334	3.042	3.014	3.166	-	-	3.921	3.825	3.840	3.959	3.874	0.631	0.664	0.530
0.0	-	-	-	-	-	-	-	-	-	-	3.698	3.763	3.715	3.731	3.672	-	-	-	-	-	-	-	-
0.2	3.940	3.856	3.788	3.749	3.876		3.233	2.852	2.813	3.392	3.778	3.681	3.670	3.721	3.771	4.000	3.956	3.898	3.870	3.810	0.573	0.549	0.648
1	4.085	4.015	3.952	3.750	4.033	-	-	-	-	-	-	-	-	-	-	-	-	-	-	-	0.657	0.577	0.509
2	2.230	1.842	2.244	1.988	1.952	1.178	1.780	1.672	1.798	1.758	3.444	3.567	3.537	3.424	3.556	3.447	3.565	3.513	3.479	3.188	0.500	0.499	0.521
3	-	-	-	-	-	-	-	-	-	-	1.571	1.566	1.497	-	-	-	-	-	-	-	0.355	0.332	0.313
4	-	-	-	-	-	-	-	-	-	-	1.108	1.093	1.073	-	-	-	-	-	-	-	-	-	-
5	0.285	0.255	0.238	0.276	0.335	0.215	0.214	0.218	0.214	0.216	0.432	0.395	0.428	0.374	0.401	1.061	1.141	1.116	1.042	1.014	-	-	-

S1-Table 7:

Cell line	WM164					WM1366					D24					HaCaT					Fibroblast		
**Transcutol (µg/mL)**																							
0.0	3.634	3.865	3.658	3.821	3.605	3.088	3.016	2.994	3.065	3.000	3.752	3.670	3.673	3.668	3.696	3.921	3.825	3.84	3.9593	3.8743	0.631	0.664	0.530
0.0	-	-	-	-	-	3.218	3.184	3.193	3.196	3.278	3.698	3.763	3.715	3.731	3.672	3.914	3.876	3.909	3.751	3.780	-	-	-
1	3.215	3.296	3.237	3.292	2.981	3.109	3.055	3.040	3.040	3.098	3.598	3.669	3.672	3.566	3.667	3.486	3.641	3.497	3.543	3.260	0.363	0.400	0.384
2	1.821	1.937	1.751	1.755	1.675	2.514	2.527	2.487	2.565	2.614	3.011	3.006	3.003	3.021	3.001	2.410	2.265	2.202	2.298	2.140	0.605	0.566	0.510
5	0.199	0.210	0.216	0.208	0.209	0.995	0.964	0.956	0.984	0.992	1.969	1.874	1.860	1.872	1.950	1.330	1.308	1.326	1.235	1.259	0.363	0.400	0.384

EXCLI Journal 2023;22:1173-1199 – ISSN 1611-2156
Received: April 03, 2023, accepted: October 11, 2023, published: November 16, 2023

S1-Table 8:

Cell line	WM164					WM1366					D24					HaCaT					Fibroblast		
**Labrafac (µg/mL)**																							
0.0	3.965	3.896	3.896	3.848	3.797	3.088	3.016	2.994	3.065	3.000	3.7517	3.67	3.6734	3.6676	3.696	3.914	3.876	3.909	3.751	3.780	0.4622	0.4576	0.4785
0.0	-	-	-	-	-	-	-	-	-	-	3.042	3.014	3.166	3.103	3.201	-	-	-	-	-	-	-	-
20	3.956	3.859	3.910	3.843	3.984	2.878	2.996	3.033	3.037	3.004	3.698	3.542	3.822	3.603	3.642	4.000	3.902	3.772	3.850	3.908	0.476	0.458	0.474
100	-	-	-	-	-	2.778	3.327	3.866	3.037	3.004	1.846	1.823	1.881	1.894	1.927	-	-	-	-	-	0.462	0.468	0.499
200	3.494	3.344	3.417	3.311	-	-	-	-	-	-	1.748	1.638	1.692	1.816	1.763	3.989	3.900	3.801	3.860	3.910	-	-	-
500	3.628	3.561	3.616	3.672	-	3.432	2.771	3.287	3.311	3.589	-	-	-	-	-	4.040	4.264	4.287	3.558	3.541	0.467	0.436	0.465

S1-Table 9: (Positive Control)

Cell line	WM164					WM1366					D24					HaCaT					Fibroblast				
**Triton X-100 (µg/mL)**																									
0.0	3.457	3.312	3.342	3.584	3.725	0.580	0.617	0.622	0.520	0.577	3.042	3.014	3.166	3.158	3.202	1.896	2.153	2.011	1.696	2.210	0.588	0.613	0.501	0.578	0.552
0.1	5.890	5.407	6.228	6.701	-	0.698	0.696	1.060	0.822	0.810	-	-	-	-	-	-	-	-	-	-	0.512	0.545	0.449	0.515	0.483
0.3	0.540	0.684	0.501	-	0.637	-	-	-	-	-	2.777	2.885	3.108	3.115	3.044	1.807	2.007	1.931	1.617	2.083	0.419	0.447	0.356	0.407	0.389
0.5	0.487	0.518	0.468	-	0.543	-	-	-	-	-	0.627	0.633	0.647	0.640	0.662	0.424	0.460	0.516	0.511	0.517	0.279	0.273	0.244	0.272	0.269
1	-	-	-	-	-	0.188	0.217	0.191	0.173	0.190	0.514	0.474	0.585	0.481	0.545	-	-	-	-	-	0.588	0.613	0.501	0.578	0.552
10	0.438	0.375	0.391	0.475	0.512	0.107	0.109	0.107	0.075	0.093	0.192	0.219	0.221	0.183	0.235	0.178	0.236	0.199	0.155	0.206	-	-	-	-	-

EXCLI Journal 2023;22:1173-1199 – ISSN 1611-2156
Received: April 03, 2023, accepted: October 11, 2023, published: November 16, 2023

Supplement 2

Raw data shows cell numbers detected by the automated cell counting machine after conducting trypan blue exclusion assay after 24 h treatment **(S2-Tables 1-4)**. Different concentrations of four tested excipients Labrasol®, Labrafil®, Transcutol®, and Labrafac® were applied on three cancer cell lines (WM164, WM1366, and D24) and two normal cells (HaCaT and primary fibroblasts).
After cell incubation for 24 h, the excipient-containing medium was removed, and cells were trypsinized using trypsin–EDTA to obtain cell suspension. 20 µl of trypan blue dye was mixed thoroughly with 20 µl cell suspension by pipetting ups and downs and loaded into the cell counting slide. Cell numbers were detected by the automated cell counting machine.

Raw tables concerning:
Figure 5: Cell viability % obtained from trypan blue exclusion assay for the excipients after 24 hr on the tested cell lines.

Table 4: IC_{50} values obtained from trypan blue, MTT, and CV (µg/mL)

(-) These concentrations have not been tested in some cell lines. Excipient concentrations were selected based on the sensitivity of the tested cell lines.

S2-Table 1:

Cell line	WM164			WM1366			D24			HaCaT			Fibroblast		
**Labrasol (µg/mL)**															
0.0	2.65	2.69	2.60	6.24	6.20	6.30	2.80	2.88	2.84	5.3	5.24	5.32	0.78	0.88	0.99
0.0	3.28	3.30	3.16	-	-	-	-	-	-	-	-	-	-	-	-
0.1	2.40	2.38	2.52	5.10	6.40	6.32	3.18	3.14	3.10	4.56	4.52	4.56	0.436	0.437	0.436
0.2	3.60	2.22	2.28	6.40	5.18	5.08	2.26	2.36	2.38	2.64	2.7	2.64	0.71	0.79	0.94
0.3	1.96	1.89	1.93	-	-	-	-	-	-	-	-	-	0.6	0.76	0.66
0.5	0.68	0.71	0.83	2.08	2.24	2.40	0.85	0.87	0.89	1.01	1.066	1.032	0.32	0.37	0.45

EXCLI Journal 2023;22:1173-1199 – ISSN 1611-2156
Received: April 03, 2023, accepted: October 11, 2023, published: November 16, 2023

S2-Table 2:

Cell line	WM164			WM1366			D24			HaCaT			Fibroblast		
**Labrafil (µg/mL)**															
0.0	2.65	2.69	2.60	6.24	6.20	6.30	2.80	2.88	2.84	5.30	5.24	5.32	0.78	0.88	0.99
0.0	3.28	3.30	3.16	-	-	-	-	-	-	-	-	-	-	-	-
1	3.88	3.88	3.70	5.04	5.00	5.10	-	-	-	5.10	5.06	5.00	0.51	0.55	0.63
2	1.90	1.85	1.91	1.24	1.32	1.34	1.72	1.61	1.55	2.48	2.44	2.54	0.27	0.23	0.26
4	0.90	0.82	0.93	0.95	0.90	0.97	0.50	0.52	0.43	0.93	1.30	0.94	-	-	-
20	-	-	-	-	-	-	0.38	0.38	0.38	-	-	-	-	-	-

S2-Table 3:

Cell line	WM164			WM1366			D24			HaCaT			Fibroblast		
**Transcutol (µg/mL)**															
0.0	2.78	2.74	2.74	6.24	6.20	6.30	2.80	2.88	2.84	5.30	5.24	5.32	0.78	0.88	0.99
0.0	3.28	3.30	3.16	-	-	-	-	-	-	-	-	-	-	-	-
5	2.78	2.74	2.74	4.76	4.72	4.62	1.97	1.94	3.90	3.48	3.56	3.54	0.44	0.53	0.58
10	1.17	1.12	1.14	4.00	3.80	3.72	1.97	1.99	1.99	1.32	1.69	1.26	-	-	-
20	0.80	0.81	0.80	0.47	0.38	0.41	0.38	0.38	0.38	0.09	0.09	0.06	0.09	0.06	0.07

S2-Table 4:

Cell line	WM164			WM1366			D24			HaCaT			Fibroblast		
**Labrafac (µg/mL)**															
0.0	2.65	2.69	2.6	2.8	2.88	2.84	2.80	2.88	2.84	5.30	5.24	5.32	0.78	0.88	0.99
100	2.83	2.89	2.85	8.66	8.6	8.48	2.28	2.48	2.56	3.94	3.98	3.92	-	-	-
200	-	-	-	6.18	6.22	6.3	1.49	1.48	1.41	4.24	4.36	4.08	1.20	1.23	1.06

EXCLI Journal 2023;22:1173-1199 – ISSN 1611-2156
Received: April 03, 2023, accepted: October 11, 2023, published: November 16, 2023

Supplement 3

Raw data shows cellular fluorescence generated by the Dihydrorhodamine 123 oxidation conducting Reactive Oxygen Species (ROS) assay **(S3-Tables 1-5)**. After 24 hours incubation with treatment solutions, medium was removed and replaced with the 100 µl of pre-warmed dying buffer (Hank's balanced salt solution (HBSS) containing 12 µM DHR dye). Cells were incubated for 30 minutes at 37 °C in a humidified 5 % CO_2 atmosphere in dark. The cellular fluorescence generated by the DHR oxidation was measured at 485 nm (OD 485) using a microplate reader (Multiskan™ FC Microplate Photometer).
Hydrogen peroxide (H_2O_2) treatment was used as positive control and dying buffer with no cells as blank control.

Raw tables concerning:
Figure 6: ROS intensity after cell exposure to increasing concentrations of excipients. Excipient concentrations are plotted from 0 to high on X axis (as the applied concentrations are varied depending on their toxicity, the applied doses are referred to as (0 to some multiple of IC50 concentration relative to the cell line and the tested excipient and ROS generation % on Y axis (ROS production results calculated as percentage of untreated control).
Figure 7: ROS generation in each tested cell line after 24 hr exposure to the excipients at their IC_{50} concentration

S3-Table 1:

Cell line	**WM164**			**WM1366**			**D24**			**HaCaT**			**Fibroblast**		
**Labrasol (µg/mL)**															
0.0	0.271	0.277	0.263	0.254	0.240	0.242	0.276	0.273	0.263	0.219	0.220	0.224	0.253	0.256	0.259
1	0.332	0.349	0.337	0.296	0.291	0.288	0.316	0.301	0.302	0.286	0.292	0.279	0.294	0.288	0.292
0.5	0.328	0.314	0.338	0.277	0.277	0.278	0.302	0.309	0.319	0.284	0.297	0.277	0.286	0.287	0.284
0.3	0.313	0.300	0.299	0.271	0.277	0.270	0.305	0.319	0.328	0.271	0.272	0.265	0.284	0.269	0.277
0.1	0.293	0.301	0.297	0.267	0.261	0.262	0.294	0.297	0.290	0.202	0.257	0.263	0.281	0.282	0.275

EXCLI Journal 2023;22:1173-1199 – ISSN 1611-2156
Received: April 03, 2023, accepted: October 11, 2023, published: November 16, 2023

S3-Table 2:

Cell line	**WM164**			**WM1366**			**D24**			**HaCaT**			**Fibroblast**		
**Labrafil (µg/mL)**															
0.0	0.276	0.274	0.251	0.243	0.236	0.222	0.273	0.251	0.224	0.211	0.219	0.220	0.257	0.248	0.222
10	0.361	0.392	0.342	0.298	0.300	0.295	0.376	0.387	0.363	-	-	-	0.276	0.268	0.232
5	0.306	0.307	0.299	0.268	0.273	0.282	0.308	0.318	0.321	0.314	0.294	0.296	0.299	0.295	0.246
2	0.293	0.306	0.298	0.253	0.259	0.259	0.302	0.303	0.305	0.289	0.299	0.276	0.309	0.300	0.243
1	-	-	-	-	-	-	-	-	-	0.280	0.281	0.283	-	-	-
0.2	0.295	0.297	0.291	0.242	0.256	0.245	0.291	0.293	0.292	-	-	-	0.281	0.282	0.283

S3-Table 3:

Cell line	**WM164**			**WM1366**			**D24**			**HaCaT**			**Fibroblast**		
**Transcutol (µg/mL)**															
0.0	0.269	0.269	0.270	0.241	0.238	0.238	0.244	0.250	0.262	0.211	0.219	0.220	0.294	0.313	0.301
50	0.338	0.326	0.326	0.286	0.273	0.287	0.310	0.321	0.312	0.279	0.292	0.270	0.315	0.327	0.306
20	0.316	0.311	0.315	0.288	0.292	0.289	0.316	0.316	0.316	0.274	0.287	0.279	0.296	0.306	0.299
10	0.299	0.295	0.294	0.258	0.264	0.277	0.305	0.305	0.308	0.262	0.266	0.268	0.290	0.293	0.281
5	0.283	0.288	0.285	0.257	0.263	0.265	0.266	0.270	0.270	0.268	0.253	0.261	0.273	0.290	0.292

EXCLI Journal 2023;22:1173-1199 – ISSN 1611-2156
Received: April 03, 2023, accepted: October 11, 2023, published: November 16, 2023

S3-Table 4:

Cell line / Labrafac (µg/mL)	WM164			WM1366			D24			HaCaT			Fibroblast		
0.0	0.244	0.271	0.275	0.225	0.250	0.252	0.267	0.269	0.271	0.219	0.220	0.224	0.226	0.244	0.237
200	-	-	-	-	-	-	-	-	-	-	-	-	0.360	0.365	0.361
100	0.287	0.314	0.305	0.295	0.290	0.288	0.345	0.338	0.331	0.317	0.313	0.378	0.360	0.353	0.363
50	0.284	0.292	0.288	0.275	0.277	0.258	0.316	0.314	0.315	0.281	0.319	0.364	0.290	0.347	0.354
10	0.247	0.295	0.292	0.253	0.273	0.265	0.289	0.306	0.308	0.310	0.326	0.284	0.223	0.277	0.286

S3-Table 5:

Cell line / H2O2 (µM) Positive control	(µg/mL)	WM164			WM1366			D24			HaCaT			Fibroblast		
0.0	0.0	0.254	0.259	0.267	0.231	0.230	0.223	0.243	0.250	0.227	0.219	0.220	0.224	0.226	0.244	0.237
0.0	0.0	0.258	0.264	0.251	0.227	0.230	0.225	0.230	0.229	0.239	-	-	-	0.253	0.256	0.259
0.0	0.0	0.260	0.261	0.257	0.223	0.228	0.223	0.292	0.225	0.223	-	-	-	0.257	0.248	0.222
0.0	0.0	0.260	0.258	0.254	0.228	0.220	0.219	0.227	0.231	0.275	-	-	-	-	-	-
800	27.0	-	-	-	-	-	-	-	-	-	0.281	0.291	0.271	0.290	0.288	0.280
500	17.0	0.315	0.301	0.296	-	-	-	-	-	-	-	-	-	-	-	-
400	13.5	0.315	0.301	0.296	0.281	0.273	0.275	0.306	0.304	0.300	0.253	0.261	0.269	0.286	0.2924	0.2854
200	6.75	0.297	0.297	0.297	0.269	0.273	0.275	0.293	0.283	0.300	0.259	0.231	0.241	0.242	0.246	0.243
100	3.4	0.302	0.301	0.251	0.265	0.258	0.268	0.294	0.304	0.297	-	-	-	-	-	-
50	1.7	0.248	0.292	0.280	0.261	0.233	0.227	0.277	0.270	0.280	-	-	-	-	-	-

EXCLI Journal 2023;22:1173-1199 – ISSN 1611-2156
Received: April 03, 2023, accepted: October 11, 2023, published: November 16, 2023

<u>Supplement 4</u>

Raw data shows cell cycle histograms with DNA content in each phase of cell cycle obtained from FACs machine according to the instructions of the manufacturer. The percentages of cells in each cell-cycle phase were analyzed using FlowJo software (v10.6.1). The PI staining was used to determine the DNA content and the cell-cycle phase distributions. Attached and floating cells were collected and fixed followed by PI staining and analyzed with FACs machine according to the instructions of the manufacturer. The percentages of cells in each cell-cycle phase were analyzed using FlowJo software (v10.6.1).

Raw tables concerning:
<u>Table 5:</u> DNA distribution (%) during cell cycle after 24-h treatment with the tested excipients

<u>Figure 8:</u> The effects of excipients on the process of the cell cycle of human melanoma and non-melanoma cells. The FACS diagram of the cell cycle. PE-CY5 channel indicates the fluorescent intensity of PI, and the Y axis indicates cell number (events).

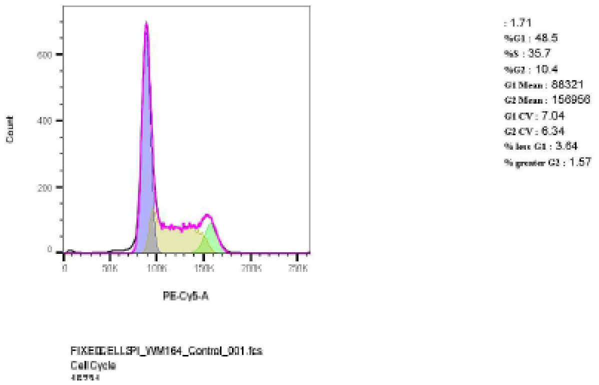

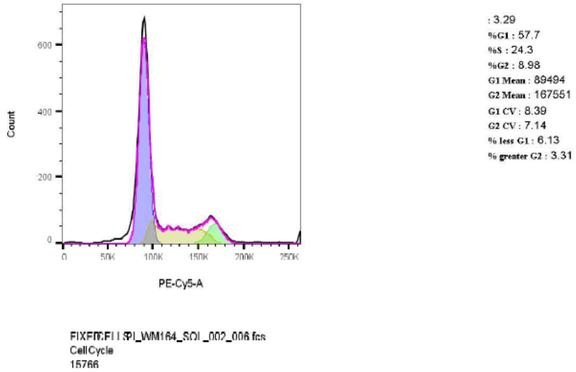

EXCLI Journal 2023;22:1173-1199 – ISSN 1611-2156
Received: April 03, 2023, accepted: October 11, 2023, published: November 16, 2023

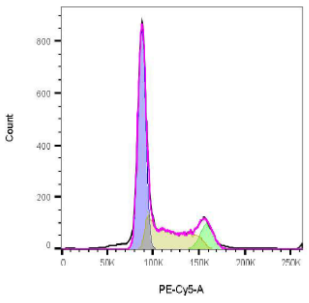

FIXEDCELLS PI_WM164_FIL_02_009.fcs
CellCycle
17532

: 2.78
%G1 : 51.7
%S : 29.2
%G2 : 10.4
G1 Mean : 87339
G2 Mean : 157940
G1 CV : 6.63
G2 CV : 6.47
% less G1 : 4.97
% greater G2 : 3.28

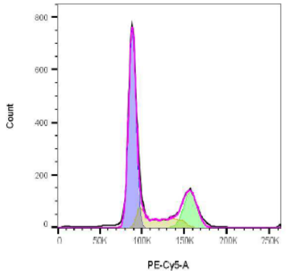

FIXEDCELLS PI_WM164_TR_05_002.fcs
Cell Cycle
15492

: 2.59
%G1 : 56.4
%S : 16.9
%G2 : 18.7
G1 Mean : 88143
G2 Mean : 156720
G1 CV : 6.99
G2 CV : 7.26
% less G1 : 3.41
% greater G2 : 2.73

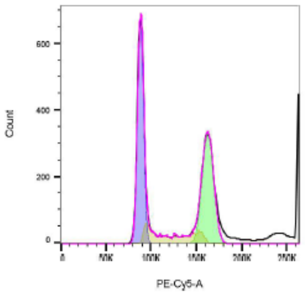

FIXEDCELLS PI_WM1366_Control_001_014.fcs
CellCycle
16093

: 16.8
%G1 : 36.4
%S : 13.2
%G2 : 32.6
G1 Mean : 87943
G2 Mean : 162206
G1 CV : 5.33
G2 CV : 5.35
% less G1 : -0.42
% greater G2 : 16.6

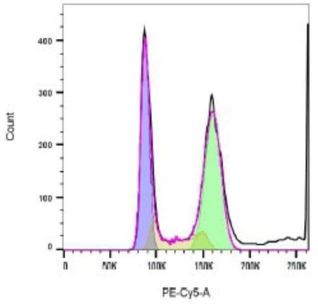

FIXEDCELLS PI_WM1366_SOL_0025_001_033.fcs
CellCycle
15587

: 16.1
%G1 : 30.8
%S : 12.7
%G2 : 38.5
G1 Mean : 87408
G2 Mean : 159894
G1 CV : 7.12
G2 CV : 7.54
% less G1 : -0.53
% greater G2 : 15.8

EXCLI Journal 2023;22:1173-1199 – ISSN 1611-2156
Received: April 03, 2023, accepted: October 11, 2023, published: November 16, 2023

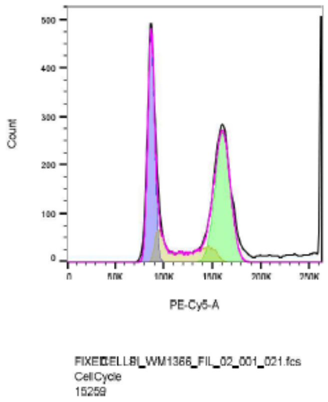

: 19.1
%G1 : 29.6
%S : 14.4
%G2 : 36.7
G1 Mean : 86517
G2 Mean : 160105
G1 CV : 5.74
G2 CV : 6.81
% less G1 : 0.72
% greater G2 : 15.3

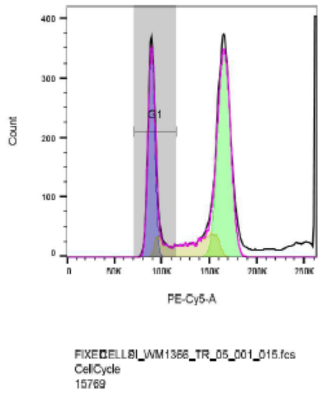

: 47.5
%G1 : 20.8
%S : 13.3
%G2 : 38.7
G1 Mean : 88885
G2 Mean : 165010
G1 CV : 5.41
G2 CV : 5.51
% less G1 : -0.011
% greater G2 : 22.9

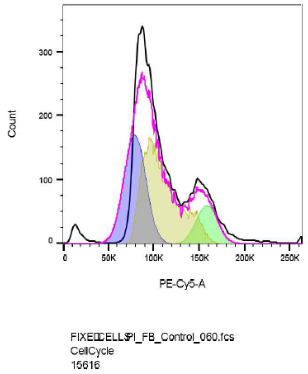

: 7.37
%G1 : 39.5
%S : 52.2
%G2 : 13.5
G1 Mean : 78949
G2 Mean : 158060
G1 CV : 21.5
G2 CV : 10.4
% less G1 : -10.2
% greater G2 : 3.21

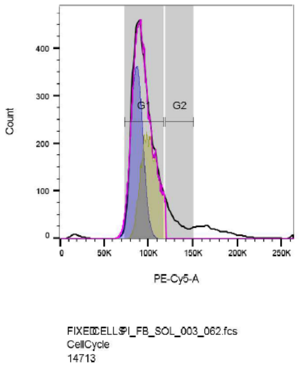

: 5.64
%G1 : 47.3
%S : 40.1
%G2 : 0
G1 Mean : 86536
G2 Mean : 119280
G1 CV : 11.3
G2 CV : 0
% less G1 : -0.69
% greater G2 : 17.1

EXCLI Journal 2023;22:1173-1199 – ISSN 1611-2156
Received: April 03, 2023, accepted: October 11, 2023, published: November 16, 2023

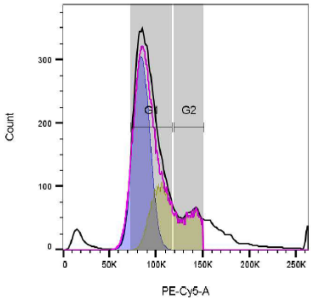

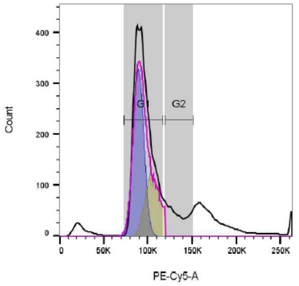

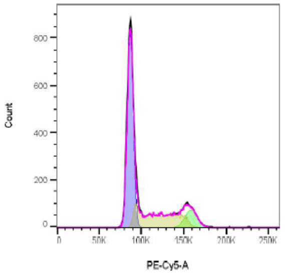

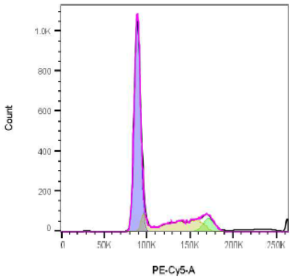

EXCLI Journal 2023;22:1173-1199 – ISSN 1611-2156
Received: April 03, 2023, accepted: October 11, 2023, published: November 16, 2023

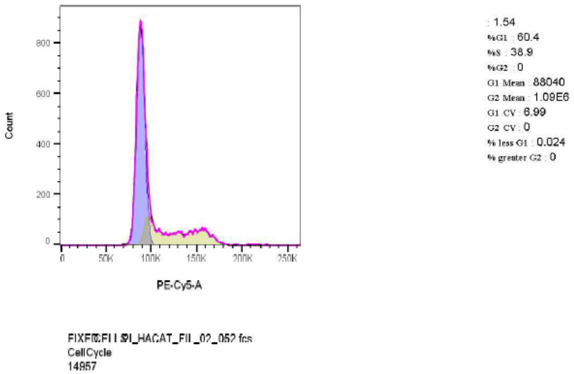

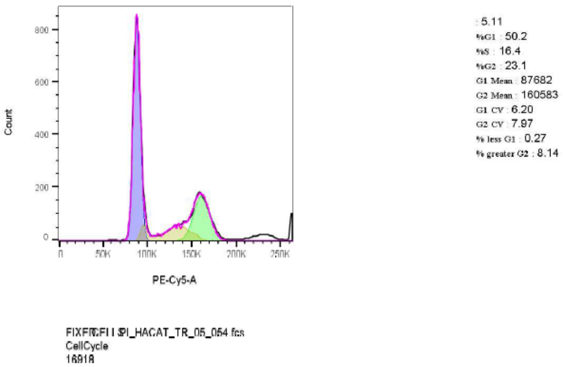

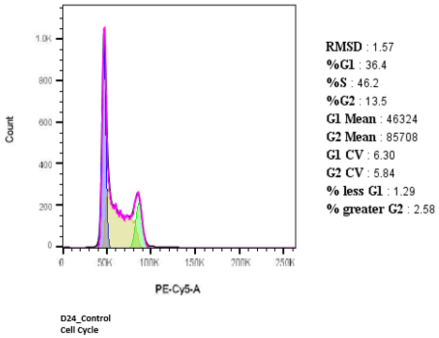

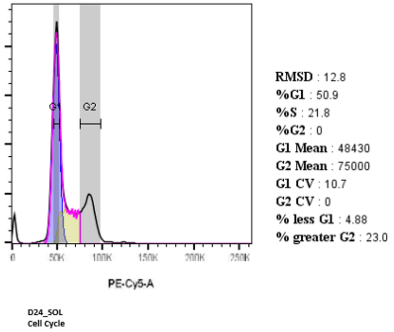

EXCLI Journal 2023;22:1173-1199 – ISSN 1611-2156
Received: April 03, 2023, accepted: October 11, 2023, published: November 16, 2023

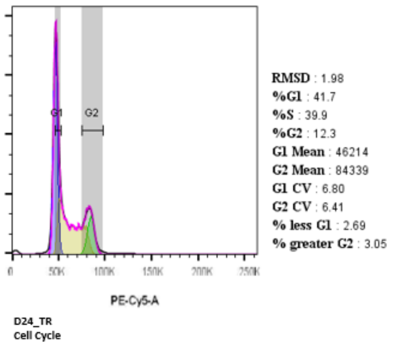

Supplement: Supplementary data [file EXCLI-22-1173-s-001.pdf]
